# Targeted drug-loaded PLGA-PCL microspheres for specific and localized treatment of triple negative breast cancer

**DOI:** 10.1007/s10856-023-06738-y

**Published:** 2023-08-02

**Authors:** Chukwudalu C. Nwazojie, John D. Obayemi, Ali A. Salifu, Sandra M. Borbor-Sawyer, Vanessa O. Uzonwanne, Chinyerem E. Onyekanne, Udom M. Akpan, Killian C. Onwudiwe, Josephine C. Oparah, Olushola S. Odusanya, Winston O. Soboyejo

**Affiliations:** 1 Department of Materials Science and Engineering, African University of Science and Technology, Km 10 Airport Road, Abuja, Nigeria; 2 Department of Mechanical and Materials Engineering, Worcester Polytechnic Institute, 100 Institute Road, Worcester, MA 01609 USA; 3 Department of Biomedical Engineering, Worcester Polytechnic Institute, 100 Institute Road, Worcester, MA 01605 USA; 4 Department of Engineering, Boston College, 140 Commonwealth Avenue, Chestnut Hill, USA; 5 Department of Biology, State University of New York, Buffalo State University, Buffalo, USA; 6 Biotechnology and Genetic Engineering Advanced Laboratory, Sheda Science and Technology Complex (SHESTCO), Abuja, Nigeria

**Keywords:** PLGA-PCL blend, Prodigiosin, Paclitaxel, EphA2, Microspheres, Triple negative breast cancer

## Abstract

The paper presents the results of the experimental and analytical study of targeted drug-loaded polymer-based microspheres made from blend polymer of polylactic-co-glycolic acid and polycaprolactone (PLGA-PCL) for targeted and localized cancer drug delivery. In vitro sustained release with detailed thermodynamically driven drug release kinetics, over a period of three months using encapsulated targeted drugs (prodigiosin-EphA2 or paclitaxel-EphA2) and control drugs [Prodigiosin (PGS), and paclitaxel (PTX)] were studied. Results from in vitro study showed a sustained and localized drug release that is well-characterized by non-Fickian Korsmeyer–Peppas kinetics model over the range of temperatures of 37 °C (body temperature), 41 °C, and 44 °C (hyperthermic temperatures). The in vitro alamar blue, and flow cytometry assays in the presence of the different drug-loaded polymer formulations resulted to cell death and cytotoxicity that was evidence through cell inhibition and late apoptosis on triple negative breast cancer (TNBC) cells (MDA-MB 231). In vivo studies carried out on groups of 4-week-old athymic nude mice that were induced with subcutaneous TNBC, showed that the localized release of the EphA2-conjugated drugs was effective in complete elimination of residual tumor after local surgical resection. Finally, ex vivo histopathological analysis carried out on the euthanized mice revealed no cytotoxicity and absence of breast cancer metastases in the liver, kidney, and lungs 12 weeks after treatment. The implications of the results are then discussed for the development of encapsulated EphA2-conjugated drugs formulation in the specific targeting, localized, and sustain drug release for the elimination of local recurred TNBC tumors after surgical resection.

**Graphical Abstract:**

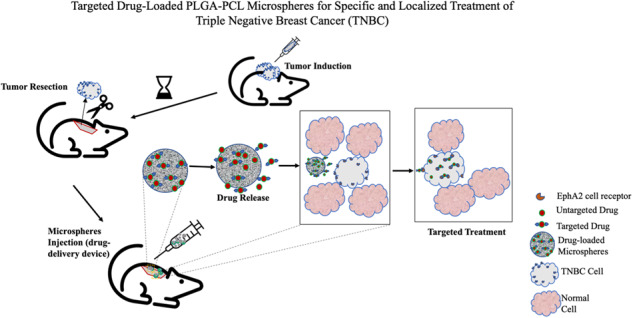

## Introduction

Cancer has been ranked as the leading cause of death with almost 10 million deaths worldwide [1]. There is about a 20% risk of getting cancer in a lifetime (before the age of 75), and a 10% risk of dying from the cancer; one in five persons will get cancer in their lifetimes and one in 10 will die from the disease [2]. Female breast cancer with an estimated 2.3 million new cases (11.7%) have surpassed lung cancer as the most diagnosed cancer followed by lung (11.4%) cancer [3].

Triple-negative breast cancer (TNBC) is a breast cancer subtype that accounts for approximately 15% of all breast cancers [4] and is characterized by the absence of the expression of the estrogen receptor (ER), progesterone receptor (PR), and human epidermal growth factor 2 receptor (HER2) [5]. TNBC subtype is the most aggressive form of breast cancer, highly metastatic with very poor prognosis as compared to other breast cancer subtypes [6]. TNBC is also associated with a high rate of recurrence [7, 8]. Effective therapies have been developed for patients with hormone receptor-positive or HER2-positive diseases, but at present chemotherapy, radiation therapy or the combination of both is the popular systemic therapy for used for the treatment of patients with TNBC [9].

Cancer therapy over the years has been characterized by unpredictability due to ineffectiveness of treatment options and side effects [10]. Chemotherapy, surgery, and radiotherapy are the most common therapeutic approaches for cancer [11–14]. Chemotherapy and radiation aims to kill the cancer cells [15–17]. Chemotherapy and radiation therapy as conventional treatment techniques are however, limited due to lack of selectivity for tumor cells over normal cells [18, 19]. These treatment methods are associated with inadequate drug concentrations to cancer cells, systemic toxicity, and the emergence of drug-resistant tumor cells [20]. Targeted therapy has become relevant due to its selectivity towards cancer cells and sparing toxicity to normal cells and aims at delivering drugs to particular receptors that are specific to the cancer tumor [21].

EphA2 is overexpressed in different cancers [22–25] and has been associated with tumor malignancy and poor prognosis [26, 27]. Higher expression of EphA2 is observed in malignant cancer-derived cell lines and advanced forms of cancer [23, 28, 29]. The different levels of EphA2 in normal cells compared with cancer cells signifies its relevance as a therapeutic target [23, 30]. Prior work by Obayemi et al. presented the results from the immunofluorescence staining of the normal breast cells (MCF 10A) and triple negative breast cancer cells (MDA-MB 231) confirmed the overexpression of EphA2 receptors on the membranes of the breast cancer cells more than those on normal breast cells [31, 32].

There are two deployable mechanisms by which EphA2 system could be used for targeted cancer treatment: by decreasing EphA2 expression or promoting EphA2 degradation, and blocking endogenous EphA2 activation [30]. Aggressive breast cancer-derived cell lines, Hs578T, MDA-435, MDA-231 and BT549, showed increased levels of EphA2 receptor compared to human mammary epithelium-derived cell lines, MCF-10A, MCF-12A and MCF-10-2 where normal levels of EphA2 are expressed [23, 29, 30]. Previous studies have reported successful targeting of EphA2 using various drug delivery devices, including liposomes, micelles, and nanoparticles [33–35]. These were achieved by conjugating the devices with an EphA2-specific monoclonal antibody that have been shown to selectively bind to EphA2-overexpressing cancer cells [36, 37]. This effort is shown to deliver anticancer drugs directly to the site of interest, which tends to increase the therapeutics effect and reduce off-target effects [38]. The goal of targeted therapy is to achieve selective tumor targeting and treatment without the killing of normal cells [21]. With the identification of novel molecular markers for cancers, strategies based on targeting these proteins have become a key component of drug delivery [39, 40].

The need to use degradable FDA-approved polymers for the encapsulation drugs have been explored by prior studies [41–48]. Drug polymer systems have been shown under in vitro and in vivo scenarios to be effective in localized cancer drug delivery in the treatment of cancer with much lower drug doses and limited side effects [49–57]. PLGA or PCL based drug delivery systems have been shown to have inefficient initial burst release [58]. In addition, because of the high hydrophobicity of PCL, it is not completely suitable as a drug delivery system [59]. PLGA and PCL blending was demonstrated to have overcome the hydrophobicity of PCL by showing increased hydrophilic properties [60]. The use of PLGA alone as a drug-loaded may result to accelerated drug release [56, 61, 62]. Hence, the need to utilize a combination of PLGA and PCL polymers in the development of drug-loaded microspheres formulation is necessary to obtain a desirable and extended drug release profile [63–65].

The paper presents the results of the experimental and analytical study of characterized targeted drug-loaded blend of FDA-approved polylactic-co-glycolic acid and polycaprolactone (PLGA-PCL) polymer microspheres in a unique proportion to encapsulated conjugated cancer drug (PGSEphA2 or PTXEphA2) for targeting and localized treatment of breast cancer. In this study, encapsulating the drugs in a carrier and delivering the drugs to the tumor site offers the advantage of reduced toxicity. EphA2 was used as for antibody-based targeting because high levels of EphA2 expression are found on the most aggressive tumor cells and in the case of this study MDA-MB-231 was used as they are a model for more aggressive and hormone-independent form of breast cancer [23, 25]. The use of PLGA-PCL blend resulted to an extended drug release which was observed for 90–120 days and corresponds with the duration of chemotherapy [66]. This paper explores the in vitro drug release kinetics, thermodynamics, and the degradation during drug release from the drug-loaded polymer blend microspheres. The in vitro and in vivo experiments showed the efficacy of the targeted drugs (PGSEphA2 and PTXEphA2) by inhibiting the growth of TNBC cells (MDA-MB-231) and preventing the regrowth of tumors in mice.

## Materials and methods

### Materials

Biodegradable poly (lactic-co-glycolic acid) (lactide: glycolide 50:50, MW 30,000–60,000) polymer that was used in study was procured from Millipore Sigma (St Louis, MO, USA). The polycaprolactone (PCL) polymer with internal viscosity of 1.0–1.3 dL/g used was procured from Durect Corporation (Cupertino, CA, USA). The EphA2 Monoclonal Antibody, N-hydroxysuccinimide (NHS), 1-ethyl-3-(3-dimethylaminopropyl) carbodiimide hydrochloride (EDC HCl), paclitaxel (PTX), and dichloromethane (DCM) were all purchased from Thermo Fisher Scientific (Waltham, MA, USA).

Prodigiosin (PGS) drug was used in this study was biosynthesized from *Serratia marcescens* as reported in our prior work [67–69]. Also, 3 kDa Amicon Ultra-4 centrifugal filter units and Amicon Pro purification system were purchased from Millipore Sigma (St Louis, MO, USA). The human triple negative breast cancer cell line (MDA-MB-231) was obtained from American Type Culture Collection (ATCC, Manassas, VA, USA), while the base growth media (L15), fetal bovine serum (FBS), penicillin/streptomycin, Dulbecco’s phosphate-buffered saline (DPBS), Alamar blue assay kit and all other cell culture reagents were purchased from Thermo Fisher Scientific (Waltham, MA, USA) unless otherwise indicated. Tissue cell culture plates, flasks, serological pipette and other supplies were procured from CELLTREAT Scientific Product (Pepperell, MA, USA).

For the in vivo study, athymic Nude-Foxn1nu strain mice with individual weights of about 17 g was procured from Envigo (South Easton, MA, USA). The surgical, restraining, handling, containment and animal study supplies were obtained from Braintree Scientific Inc. (Braintree, MA, USA). The animal protocol used for this study was approved by the Institutional Animal Care and Use Committee (IACUC) at Worcester Polytechnic Institute (IACUC docket #19-113) and was carried out in accordance with the guideline of the use of laboratory animals outlined by NIH.

### Drug conjugation with EphA2

EphA2-drug conjugation was achieved by the one-step conjugation approach that involves reacting lysine ε-amino groups of an antibody and a drug possessing amine-reactive group to create amide bonds [70]. Each free drug (prodigiosin and paclitaxel) was dissolved in DMSO to make the concentration of 5 mg/ml. This was followed by the addition of EDC (with masses that gives the same molarity as the parent drug) dissolved in double deionized water (ddH_2_O) whilst shaking. NHS (with masses that gives the same molarity as the parent drug) dissolved in ddH_2_O is subsequently added to the resulting reaction mixture at 4 °C for 1 h. Finally, 0.5 mg/ml of EphA2 antibody (in ddH_2_O and DMSO) was added to the reaction mixture for 4 h to tether the EphA2 to the drugs. In each case of the drug used, a corresponding targeted PTXEphA2 or PGSEphA2 drug is formed.

### Fabrication of drug-loaded microspheres

EphA2-conjugated drug (PTXEphA2 or PGSEphA2), unconjugated drug (PTX or PGS) is encapsulated each in polymer microspheres made from blend of PLGA and PCL polymer using single emulsion solvent evaporation technique. In this work, a 3% (w/v) poly(vinyl alcohol) (PVA) emulsifier was prepared with deionized water (solution A). Four hundred microgram polymer blends of PLGA and PCL (in a ratio of 4:1) were dissolved in 2 ml of dichloromethane (DCM). The PLGA-PCL polymer blend solutions were then vortexed at 1000 rpm for 5 min to obtain a resulting homogenous mixture. Thereafter, 0.2 ml of 5 mg/ml PTX, PTXEphA2, PGS, or PGSEphA2, were separately and freshly prepared with DCM. To each of the polymer blend, a prepared drug constituent/solution was mixed vigorously via homogenization (Solution B). In each case of the drug formulations prepared, Solution B was added dropwise into an aqueous solution of 3% (w/v) PVA (Solution A-surfactant) while homogenizing with an UltraTurrax T10 homogenizer (IKA, Wilmington, NC) at 22,000 rpm continuously for 5 min.

To each formulation prepared, the resulting solution is stirred with a magnetic stirrer for a period of 2 h at 600 rpm. After which the stirred solution was centrifuged at 4500 rpm for 10 min to obtain the pellets microspheres (which is the mixture of the blend of polymer and the corresponding drug). Usually, excess PVA in the pellets was removed by washing four times in the presence of tap water through centrifugation for 10 min at 4500 rpm. Finally, the drug-loaded microspheres obtained were frozen at −80 °C followed by lyophilization for 48 h (Benchtop SLC Freeze Dryer, Virtis, Warminster, PA, USA). To obtain a control sample, a non-drug-loaded PLGA-PCL microspheres (control) was prepared adopting the same approach but without the addition of any drugs. The final product of drug-loaded microspheres is freeze-dried and stored at −20 °C until needed.

### Physicochemical characterization of drug-loaded microspheres

The physicochemical of the conjugated drug with the morphological, and thermal properties of the drug-encapsulated microspheres were characterized using Fourier-transform infrared spectroscopy (FTIR), nuclear magnetic resonance (NMR), dynamic light scattering (DLS), scanning electron microscopy (SEM), thermogravimetric analysis (TGA), and differential scanning calorimetry (DSC).

FTIR was used to identify the chemical bonds in the EphA2-conjugated drugs (PGSEphA2 and PTXEphA2) and the drug-encapsulated microspheres using an FTIR spectrometer (IRSpirit, Shimadzu, Kyoto, Japan) with an average of 128 scans at 2 cm^−1^ resolution over 500–40002 cm^−1^ wavenumber range. NMR spectroscopy was used to analyze the structure of the non-loaded and drug-loaded PLGA-PCL microspheres formulations. This achieved with a Bruker Advance 400 MHz (Bruker, Billerica, MA, USA) and by dissolving 10 mg of the PLGA-PCL microspheres in 1 ml of deuterated chloroform (CDCl_3_). The Bruker’s TopSpin Software package (v 4.1.1) was used to obtain and analyze the H-NMR spectra of the different microspheres’ formulations.

DLS analysis was undertaken to determine the hydrodynamic diameters and polydispersity indices (PDI) of the drug-loaded and control microspheres using a Malvern Zetasizer Nano ZS (Malvern Instruments, Malvern, UK). SEM was used to characterize the morphologies of the microspheres. First, the freeze-dried microspheres were mounted on double-sided tape on an aluminum stub before they were sputter-coated with 5 nm of gold and visualized using a field emission SEM (7000F FE-SEM, JEOL, MA, USA). The mean diameters of the microspheres were also determined from the SEM micrographs using the image J analysis package.

The thermal properties [decomposition temperature, glass transition temperature (Tg), and melting temperature (*T*_m_)] of the drug-loaded microspheres and their controls were determined using a TGA analyzer (TG 209 FI Libra, NETZSCH, Selb, Germany) and DSC calorimeter (DSC 214 Polyma with IC40, NETZSCH, Selb, Germany). TGA thermograms were obtained for the samples between 20 and 900 °C with a constant heating rate of 20 K/min under nitrogen gas. DSC curves were obtained using 10 mg (±0.5 mg) mass of each drug-loaded microsphere and the control microsphere (PLGA/PCL) in sealed aluminum pans. A reference sample pan containing nothing, and the resulting samples were run under a heat/cool/heat cycle from −10 to 200 °C (depending on the decomposition temperature obtained from the TGA). Heating and cooling rates were carried out, respectively, at 10 °C/min and −10 °C/min, under a steady supply of nitrogen gas at 20 ml/min.

Finally, the percentage yield of all prepared formulations including the control microspheres is quantified using the formula below:

1 $$\% \,{{{\mathrm{Yield = }}}}\frac{{{{{\mathrm{Actual}}}}\,{{{\mathrm{yield}}}}}}{{{{{\mathrm{Theotrical}}}}\,{{{\mathrm{yield}}}}}} \times 100\%$$

### In vitro studies

#### In vitro drug release

The amount of drug successfully loaded into the polymer microspheres was determined before the drug release studies. To achieve this, 10 mg of the freeze-dried drug-loaded microsphere samples with different drug loading (PGS, PGSEphA2, PTX, or PTXEphA2) was dissolved in 1 ml of DCM and vortexed to achieve a homogenous solution. The absorbance values were then obtained using a UV–Vis spectrophotometer (UV-1900, Shimadzu, Tokyo, Japan) at wavelengths of 235 nm for PTX and PTXEphA2, and at 535 nm for PGS and PGSEphA2 drugs. Absorbance readings from non-loaded microspheres were deducted from those of the drug-loaded microspheres to obtain corrected absorbance values. Standard calibration curves were constructed by dissolving known concentrations of PGS, PGSEphA2, PTX, and PTXEphA2 in DCM [68] used to estimate the concentration of the drugs loaded in the microspheres using the corrected absorbance values.

In vitro drug release studies were used to characterize the release profiles of the drugs (PGS, PGSEphA2, PTX, and PTXEphA2) and to determine the kinetics and thermodynamics of drug release from the microspheres. The release of the drugs (PGS, PGSEphA2, PTX, and PTXEphA2) from the drug-loaded polymer microspheres were studied experimentally at physiological (37 °C) and hyperthermic temperatures (41 and 44 °C) over a 3-month period. For each drug-loaded microsphere formulation, 10 mg of the microspheres was suspended in 10 ml of PBS (pH 7.4) in triplicate using a 15 ml screw-capped centrifuge tubes. The tubes were then placed in an incubator shaker (Innova 44 Incubator, Console Incubator Shaker, New Brunswick, NJ, USA) that was maintained at 37 °C and shaken at 60 rpm.

At time intervals of 24 h over a period of 3 months, the tubes were centrifuged at 3000 rpm for 5 min and 1 ml aliquots of the supernatant were taken for absorbance measurements using a UV–Vis spectrophotometer (UV-1900, Shimadzu, Tokyo, Japan) at wavelengths of 235 nm for PTX and PTXEphA2, and at 535 nm for PGS and PGSEphA2 drugs. The tubes were replenished with 1 ml of fresh PBS to maintain sink condition and returned to the incubator shaker until the next sampling time. The concentrations of the released drugs from the drug-loaded microspheres were then interpolated from their respective drug standard curves to obtain time-dependent drug release. Under in vitro conditions, changes in the morphologies microstructure, and the degradation and release rates of the drug-loaded microsphere, were also characterized. Finally, the drug encapsulation efficiency (DEE) and the drug loading efficiency (DLE) were measured using the same approach and equations to those reported in our prior work [65].

#### Drug release kinetics

The kinetics of the drugs released from the drug-loaded PLGA-PCL microparticles were determined by fitting the release data to Zeroth order kinetics, First Order Kinetics, Higuchi Model, and Korsmeyer–Peppas model. In the case of the Zeroth order kinetics, the drug release rate from the drug-loaded microspheres is independent of concentration. Therefore, the relationship between % cumulative drug release versus time was plotted using the equation below.

2 $${{{Q}}}_{{{\mathrm{t}}}} = {{{Q}}}_0 + {{{K}}}_{0 \cdot }{{{t}}}$$

where *Q*_t_ represents the cumulative amount of drug released in time ‘*t*’, *Q*_0_ represents the initial amount of drug in the solution, *K*_0_ is the zeroth order release constant, and ‘*t*’ is the time (*h*).

The first order equation that describe the drug release kinetics is given below.

3 $${{{\mathrm{log}}}}\,Qt = {{{\mathrm{log}}}}\,Q_0 + Kt/2.303$$

From Eq. (3), a plot of the log % cumulative drug release versus time. where *K* is the first order release constant. kinetics is often observed during the dissolution of water-soluble drugs in porous matrices.

In the case of Higuchi model, the relationship between the % cumulative drug release versus the square root of time (√*t*) is plotted (Eq. 4).

4 $${{{Q}}}_t = {{{Q}}}_0 + {{{K}}}_{{{\mathrm{H}}}}\,t^{1/2}$$

where *K*_H_ is the Higuchi release constant. In cases where the amount of drug changes from *Q*_0_ to *Q*_t_, the Higuchi model is expressed as:

The Korsmeyer–Peppas model describes is described in Eq. 5. Equation of the rate of drug release of active agents from the drug-loaded microspheres is represented in Eq. (5).

5 $$\frac{{Q_t}}{{Q_\infty }} = kt^n\,{{{\mathrm{and}}}}\,{{{\mathrm{ln}}}}\left( {\frac{{Q_t}}{{Q_\infty }}} \right) = {{{\mathrm{ln}}}}\,k + n\,{{{\mathrm{ln}}}}\,t$$

where *Q*_∞_, *Q*_*t*_, *t*, *k* and *n* represent the cumulative amount of drug released at equilibrium, the quantity of drug release in time *t*, period of drug release, release rate constant and release exponent, respectively.

Considering the four models, the one that provides the best *R*^2^ value is selected as the kinetic model with the best fit that truly represents and characterize the drug release data.

#### Thermodynamics of drug release

The thermodynamic parameters associated with drug release from the drug-loaded PLGA-PCL microspheres formulations were determined from the drug release and kinetics data. These include: the activation energy (E_a_), enthalpy change (Δ*H*), change in Gibbs free energy (Δ*G*), and entropy change (Δ*S*). These were determined using methods from our prior studies [65]. First, the *E*_a_ was determined from the Arrhenius equation (Eq. 6) from the slope of the plot of ln *k*_*t*_ versus 1/T.

6 $$k_t = D_fe^{ - \frac{{E_a}}{{RT}}}$$

7 $$lnk_t = lnD_f - \frac{{E_a}}{R}\frac{1}{T}$$

The enthalpy change (∆*H*) and entropy change (∆*S*) were determined from the Eyring equation (Eq. 9) from the slope and intercept, respectively, from the plot of *ln k*_*t*_ versus 1/*T*.

8 $$\ln \frac{{k_t}}{T} = \frac{{\Delta H}}{R}\frac{1}{T} + \ln \frac{{\kappa k_B}}{h} + \frac{{\Delta S}}{R}$$

Finally, the change in Gibbs free energy (∆*G*) was calculated from Eq. 9:

9 $$\Delta G = \Delta H - T\Delta S$$

where *R* is the universal gas constant 8.314 Jmol^−1^ K^−1^, *T* is the absolute temperature in Kelvin, *k*_t_ is the thermodynamic equilibrium constant, *E*_a_ is the activation energy, ∆*S* is the entropy change, ∆*H* is the enthalpy change, *κ* is the transmission coefficient, *k*_B_ is the Boltzmann constant, 1.38065 × 10^−23^ m^2^ kgs^−2^ K^_1^ and *h* is Planck’s constant, 6.626 × 10^−4^J s.

#### In vitro cell viability studies

The human triple-negative breast cancer cell line (MDA-MB-231) was cultured in a complete culture medium (L-15+) which includes Leibovitz’s L15 medium supplemented with 10% fetal bovine serum (FBS), and 1% penicillin/streptomycin. The cells were monitored in an incubator at 37 °C in the absence of CO_2_ and were harvested at 70–80% confluence using 0.25% trypsin-EDTA solution. The cells were subsequently subculture into new T75 flasks. The goal of this in vitro experiment is to explore the effectiveness of the targeted drug-loaded microspheres in reducing cell viability via targeted and localized delivery of breast cancer cells.

Alamar blue assay was used to investigate in vitro cell viability and cytotoxicity using methods described in our recent studies [64, 65, 71]. This was used to ascertain the possible effects of drug-induced toxicity on triple-negative breast cancer (MDA-MB-231) cells. At 5- 7 cell passages, 10^4^ cells/well were seeded in 24-well plates (*n* = 4) in L-15^+^ culture medium and incubated overnight at 37 °C. Subsequently, the culture medium in each well was replaced with 1 ml of culture medium containing 1.0 mg/ml of the drug-loaded microspheres and their controls. The microspheres were exposed to UV light for 3 h to sterilize them prior to the cell viability studies.

At predetermined time points following the treatment of the cells with drug-loaded microspheres (0h, 6h, 24h, 48h, 72h, and 96 h), the culture medium was replaced with culture medium containing 10% Alamar blue reagent and incubated at 37 °C and 5% CO_2_ for 3 h. 100 μl aliquots were then transferred into black opaque 96-well plates and the fluorescence intensities were measured (excitation/emission: 544/590 nm) using a 1420 Victor3 multilabel plate reader (Perkin Elmer, Waltham, MA, USA). The percentage alamar blue reduction was calculated from Eq. (10).

10 $$\% \,{\rm{Alamar}}\,{\rm{Blue}}\,{\rm{Reduction}} = \frac{{FI_{\rm{sample}} - FI_{10\% \rm{AB}}}}{{FI_{100\% R} - FI_{10\% {\rm{AB}}}}} \times 100$$

where *FI*_Sample_, *FI*_10%AB_ and *FI*_100%*R*_ represent the fluorescence intensity of treated cells, fluorescence intensity of 10% alamar blue, and fluorescence intensity of 100% reduced alamar blue, respectively.

#### Flow cytometry analysis

Flow cytometry analysis was carried out to determine the mechanism of cell death associated with the drugs released from the microspheres. First, about 1 × 10^5^ cells were cultured in triplicate in six well tissue culture plate. These cells were then treated with the drug-loaded microspheres and non-loaded microspheres for 24 h were harvested via trypsinization, centrifuged, and then stained for apoptosis using the Annexin V-FITC apoptosis staining/detection kit (Abcam, Cambridge, MA) according to the manufacturer’s instructions. This was done by centrifuging the tripsinized cells and centrifuging the cells at 900 rpm for 5 min to obtain cell pellets. The resulting cells were then resuspended in 500 μl of binding buffer followed by the addition of 5 µl of Annexin V-FITC and 5 μl of propidium iodide in the dark for 15 min at room temperature. Flow cytometry analysis was then performed on the stained cells using a BD FACSAria Fusion flow cytometer.

### In vivo animal studies

Similar method of in vivo animal study used in this study is similar to those in our previous studies [65, 71]. Thirty 4-week-old healthy immunocompromised female athymic nude-Foxn1nu mice that weigh ~17 g each were purchased from Envigo (South Easton, MA, USA). The nude mice were allowed to acclimatize to the new surrounding for few days prior to when the subcutaneous xenograft triple negative breast cancer is induced. At about 6-weeks old, we used the mice to investigate the extent to which breast tumor regrowth or locoregional recurrence can be treated and prevented after surgical resection using the drug-loaded PLGA-PCL. The animal protocol used for this study was approved by the Institutional Animal Care and Use Committee (IACUC) at Worcester Polytechnic Institute (IACUC docket #19-113) and was carried out in accordance with the guideline of the use of laboratory animals outlined by NIH.

The treatment groups were based on the drug-loaded formulations that will be implanted into the region where subcutaneous xenograft tumor removed. The thirty mice were randomly divided into six groups of five mice each. The individual groups were based on the different microsphere formulations: PLGA-PCL_PTX, PLGA-PCL_PTXEphA2, PLGA-PCL_PGS, PLGA-PCL_PGSEphA2, negative control (PLGA-PCL) and positive control (without any microsphere). The control groups include those implanted the non-loaded microsphere (PLGA-PCL) and those mice without microspheres (used as the baseline for evaluation to compare with the drug-loaded microspheres’ group).

Few days after the mice arrived and acclimatized, subcutaneous TNBC tumors were induced in the interscapular region on the mice via injection of 5 × 10^6^ MDA-MB-231 cells harvested from the in vitro cell culture. The subcutaneous tumor sites and the well-being of the mice were closely observed for a period of 2 weeks until they are large enough for tumor resection and drug microsphere implantation. Beyond the tumor growth stage, the body weight of the mice was measured to observe for any sign of weight loss, signs of infections, and abnormalities. The length and width of each mouse tumor was measured using digital Vernier caliper. The xenograft tumor volume after two weeks of induction were estimated using the same approach as reported in prior work [72, 73].

To allow sufficient tumor regrowth and to demonstrate effectiveness of the targeted and localized drug release from the drug-loaded microspheres, partial surgical removal of subcutaneous tumors (~90%) were carried out. Thereafter, 200 mg/ml of drug-loaded microsphere of each formulation and negative control (PLGA-PCL) were implanted at the site of the surgical resection immediately after the removal of the xenograft induced tumor. Localized cancer drug release was monitored for each group for a period of 12 weeks checking for any tumor regrowth and metastasis effect. Body weight measurements were also taken every 3 days up to check for any possible weight loss/gain. At the end of the 12-week duration, all the mice were euthanized, and the subcutaneous tumors, lungs, kidney, and liver were all carefully excised, labeled, and preserved in a cryo sample container in a liquid Nitrogen tank for further ex vivo studies using hematoxylin and eosin (H&E) staining to check for any toxicity and metastasis.

### Ex vivo studies

Histopathology evaluation using hematoxylin and eosin (H&E) staining kits (Vector Laboratories, Newark, CA) of the lungs, liver, kidney, and some cases of metastasized tumor were carried out. The excised frozen organs/tissue were embedded in optimum cutting temperature (OCT) compound and processed in a cryostat (Leica CM3050 S Research Cryostat, Leica Biosystems Inc., Buffalo Grove, IL, USA). Five micromoters thicknesses of each of the tissue an organs were sectioned along the longitudinal axis using a comparable technique from our recent studies [64, 65, 71]. These sections were placed on glass slides and allowed to stay overnight in the −80 °C freezer. Prior to the H&E staining, the samples on the glass slides are allowed to thaw and are fixed in 80% Methanol at 4 °C for 5 min, PBS for 10 min, rinsed in deionized water. The resulting samples were then stained with hematoxylin and eosin (H&E) following the methods as recommended by Vector Laboratories. The stained slides were examined using a 20 × objective lens TS100F Nikon light microscope (Nikon Instruments Inc., Melville, NY, USA) that was coupled with a DS-Fi3 C digital camera.

### Statistical analysis

The results are presented as mean ± standard deviation for three independent trials (*n* = 3), unless otherwise indicated. One-way ANOVA was used to analyze the differences in drug release from the various microsphere formulations at different temperatures, whereas two-way ANOVA was used to analyze the differences in cell viability after treatment with the drug-loaded microsphere formulations. Post hoc Tukey multiple comparison tests were used to identify the statistically significant groups. The analyses were carried out using the SPSS package (v28) and statistical significance was set at *p* < 0.05.

## Results

### Targeted drugs microspheres and characterization

#### EphA2-conjugated drugs

**Fig. 1:**
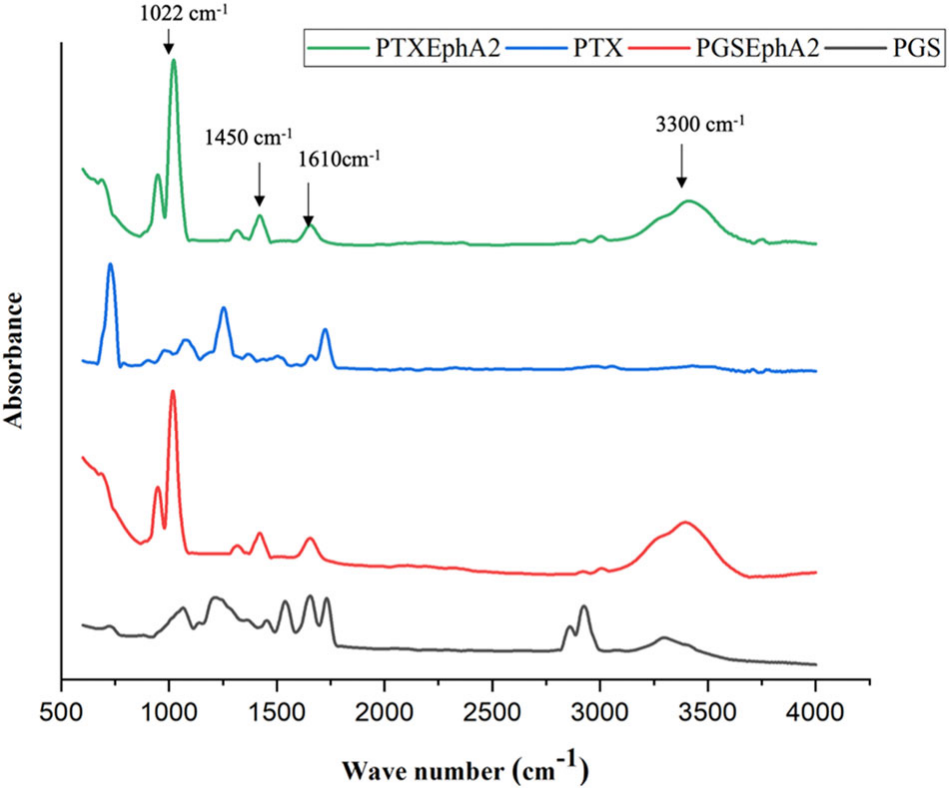
FTIR spectra of conjugated drugs (PGS and PTX) and non-conjugated drugs (PGSEphA2 and PTXEphA2)

The EphA2-drug-conjugated spectra (Fig. 1) revealed the presence of characteristic bands of –NH_2_ (3300 cm^−1^). The conjugated drugs also exhibited typical amide (covalent or peptide) bond signatures at around 1610 cm^−1^ that has been reported [74].

#### Drug-encapsulated PLGA-PCL microspheres

Table 1 shows percentages of yield of all the prepared formulations of drug-loaded microspheres. Percentage yield from the theoretical and actual yield of the microspheres were calculated.

**Table 1:** Yield Percentage of PTX-based drug-loaded and PGS-based drug-loaded formulations

Formulations	Theoretical yield (mg)	Actual yield (mg)	%Yield
PLGA-PCL_PGS	400	337.0	84.3
PLGA-PCL_PGSEphA2	400	258.0	64.5
PLGA-PCL_PTX	400	325.5	81.4
PLGA-PCL_PTXEphA2	400	200.0	50.0

**Fig. 2:**
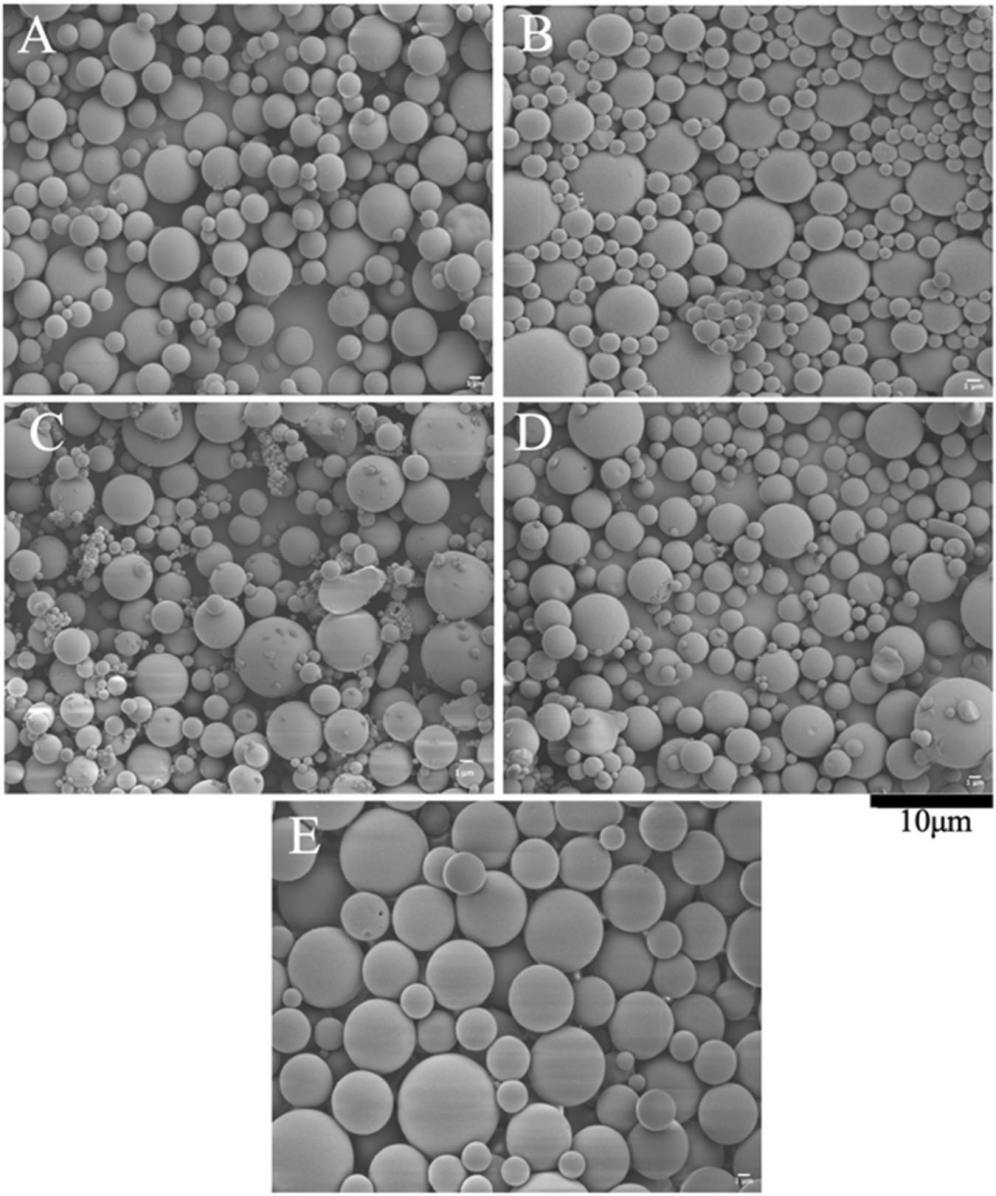
SEM micrographs of **A** PLGA_PCL **B** PLGA_PCL-PGS **C** PLGA_PCL-PGSEphA2 **D** PLGA_PCL_PTX **E** PLGA_PCL_PTXEphA2 microsphere formulations

**Fig. 3:**
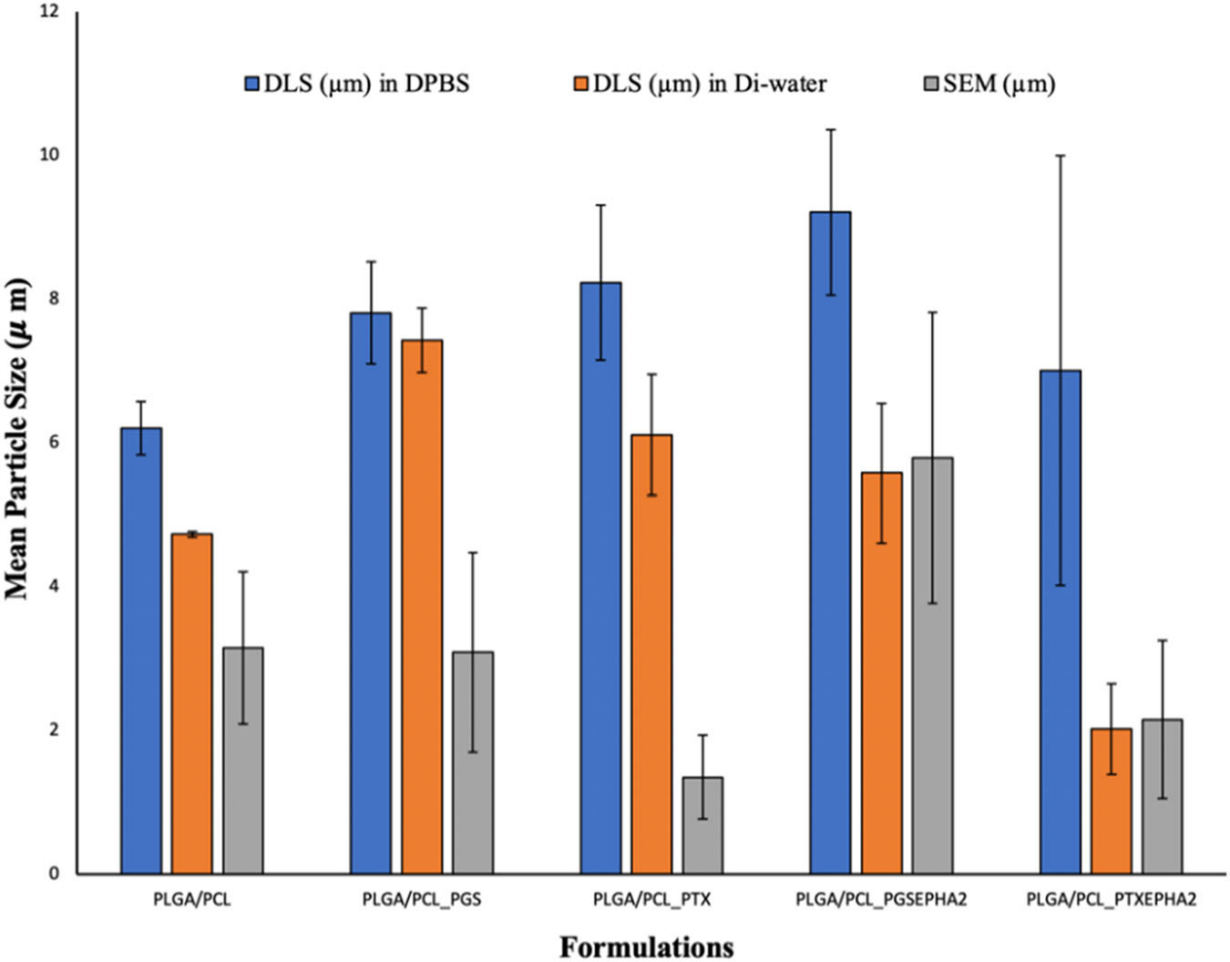
Mean particle sizes in (mm) of targeted drug-encapsulated microspheres system

The morphological and structure of the drug-loaded microspheres with the control microspheres that were imaged with the SEM are presented in Fig. 2A–E. These SEM micrographs represent (A) PLGA-PCL, (B) PLGA-PCL_PGS, (C) PLGA-PCL_PGSEphA2 (D) PLGA-PCL_PTX (E) PLGA-PCL_PTXEphA2. Results from image analyses showed that the mean particle sizes of the microparticles formulations were between 1.35 and 5.79 μm (Fig. 3).

Interestingly, the results of the hydrodynamic diameter of the drug-loaded microparticles in DPBS and DiH_2_O from the DLS (Fig. 3) are in the ranges of 6.2–9.2 μm, and from 2.02 to 7.42 μm, respectively.

**Fig. 4:**
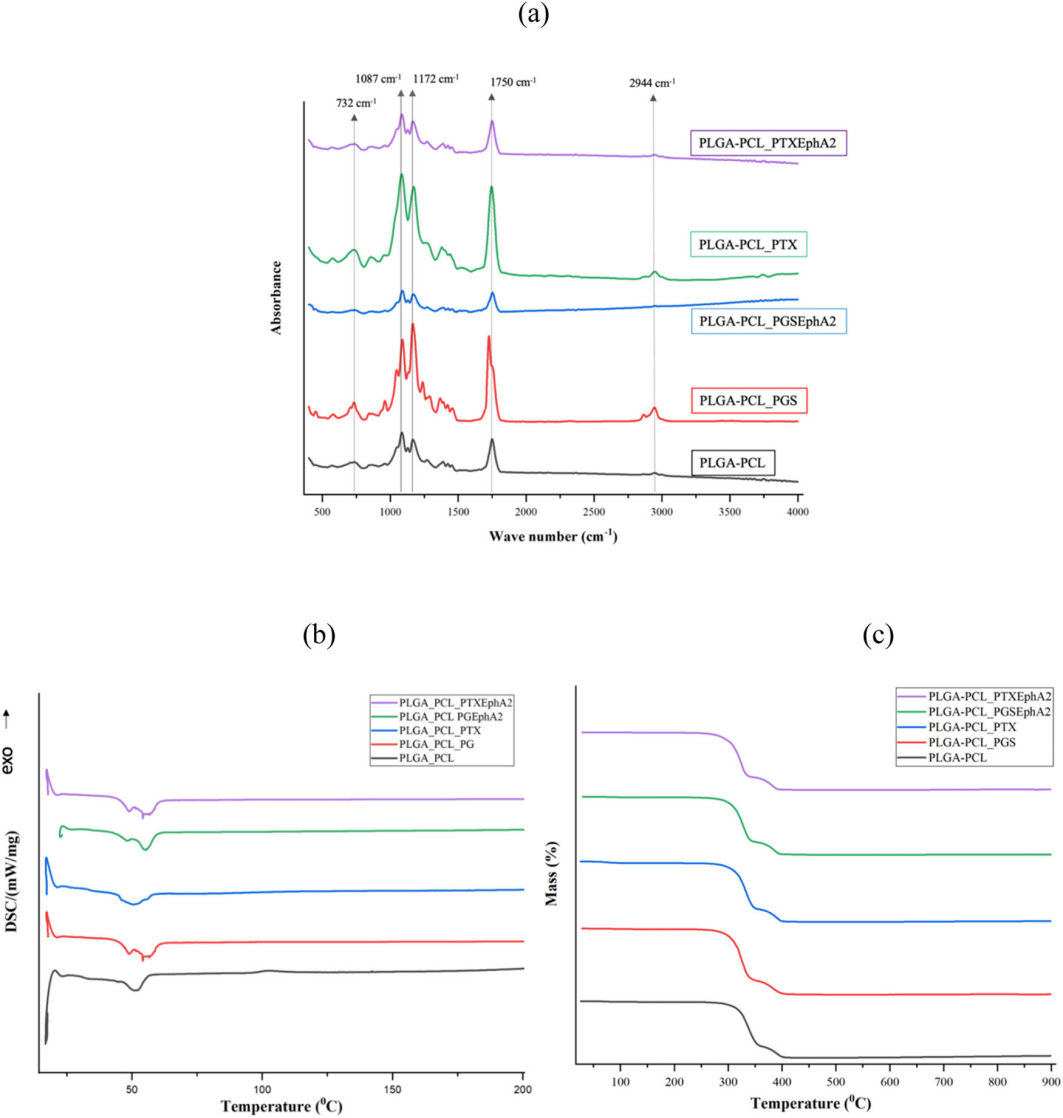
**a** FTIR spectra of PLGA-PCL microspheres and drug-loaded microspheres (PLGA-PCL_PGS, PLGA-PCL_PGSEphA2, PLGA-PCL_PTX, PLGA-PCL_PTXEphA2), **b** DSC thermographs of PLGA-PCL and PLGA-PCL drug-loaded microspheres (PLGA-PCL_PGS, PLGA-PCL_PGSEphA2, PLGA-PCL_PTX, PLGA-PCL_PTXEphA2) and **c** Thermogravimetric analysis (TGA) curves of PLGA-PCL and PLGA-PCL drug-loaded microspheres (PLGA-PCL_PGS, PLGA-PCL_PGSEphA2, PLGA-PCL_PTX, PLGA-PCL_PTXEphA2)

**Fig. 5:**
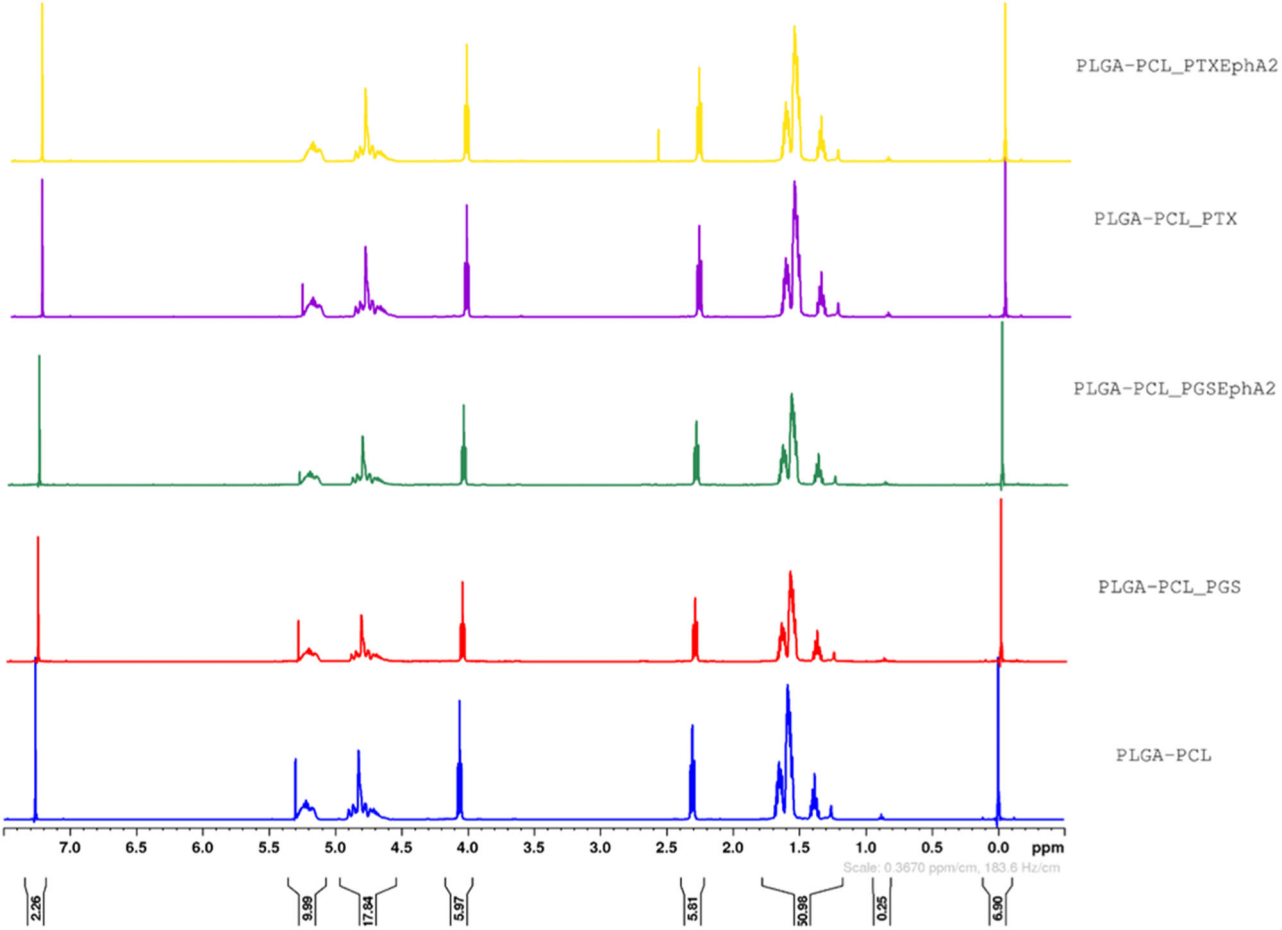
^1^HNMR spectrograms of PLGA-PCL and drug-loaded PLGA-PCL microspheres formulations

The results of the physicochemical characterization of the drug-loaded nanoparticles carried out using FTIR, NMR, TGA, DSC are presented in Figs. 4 and 5. This enables us to explore the chemical and physical properties, that include their composition, and thermal stability of the drug-loaded systems. The FTIR spectra obtained for the drug-loaded microspheres in Fig. 4a represents those of PLGA-PCL, PLGA-PCL_PGS, PLGA-PCL_PTX, PLGA-PCL_PGSEphA2 and PLGA-PCL_PTXEphA2. While the DSC and TGA results are presented in Fig. 4b, c, respectively.

The glass transition temperature, *T*_g_, melting temperature, delta heat capacity and decomposition temperature were tabulated (Table 2). This parameters are necessary to unravel the thermal properties of the drug-loaded micropheres systems.

**Table 2:** Glass transition temperatures (Tg), endothermic peak temperatures and change in heat capacity (∆Cp) values for PLGA-PCL and PLGA-PCL drug-loaded (PLGA-PCL, PLGA-PCL_PGS, PLGA-PCL_PGSEphA2, PLGA-PCL_PTX and PLGA-PCL_PTXEphA2) microspheres

Formulations	Glass transition temperature (*T*_g_) (°C)	Endothermic peak (°C)	Delta heat capacity (∆*C*_p_) J/(g K)	Decomposition temperature (°C)
PLGA_PCL	44.8	49.1	1.8	325.2
PLGA-PCL_PGS	52.2	54.2	0.5	304.3
PLGA-PCL_PGSEphA2	50.6	55.5	0.2	313.7
PLGA-PCL_PTX	46.8	50.6	0.7	318.8
PLGA-PCL_PTXEphA2	55.0	57.9	0.3	320.5

The HNMR results obtained for the different drug-loaded systems are presented in Fig. 5. There were three peaks in the PLGA spectrum at *δ* = 5.299 (H, H_a_), 4.8 (2H, H_c_), and 1.55 (3H, H_b_), respectively. Figure 5 also shows four peaks of proton signals in the PCL spectrum at *δ* = 4.06 (2H, Hd), 2.3 (2H, Hg), 1.6–1.66 (2H, He), and 1.39 (2H, Hf) [75].

### Drug release kinetics and thermodynamics with degradation of drug-encapsulated microspheres

#### In vitro drug release

**Fig. 6:**
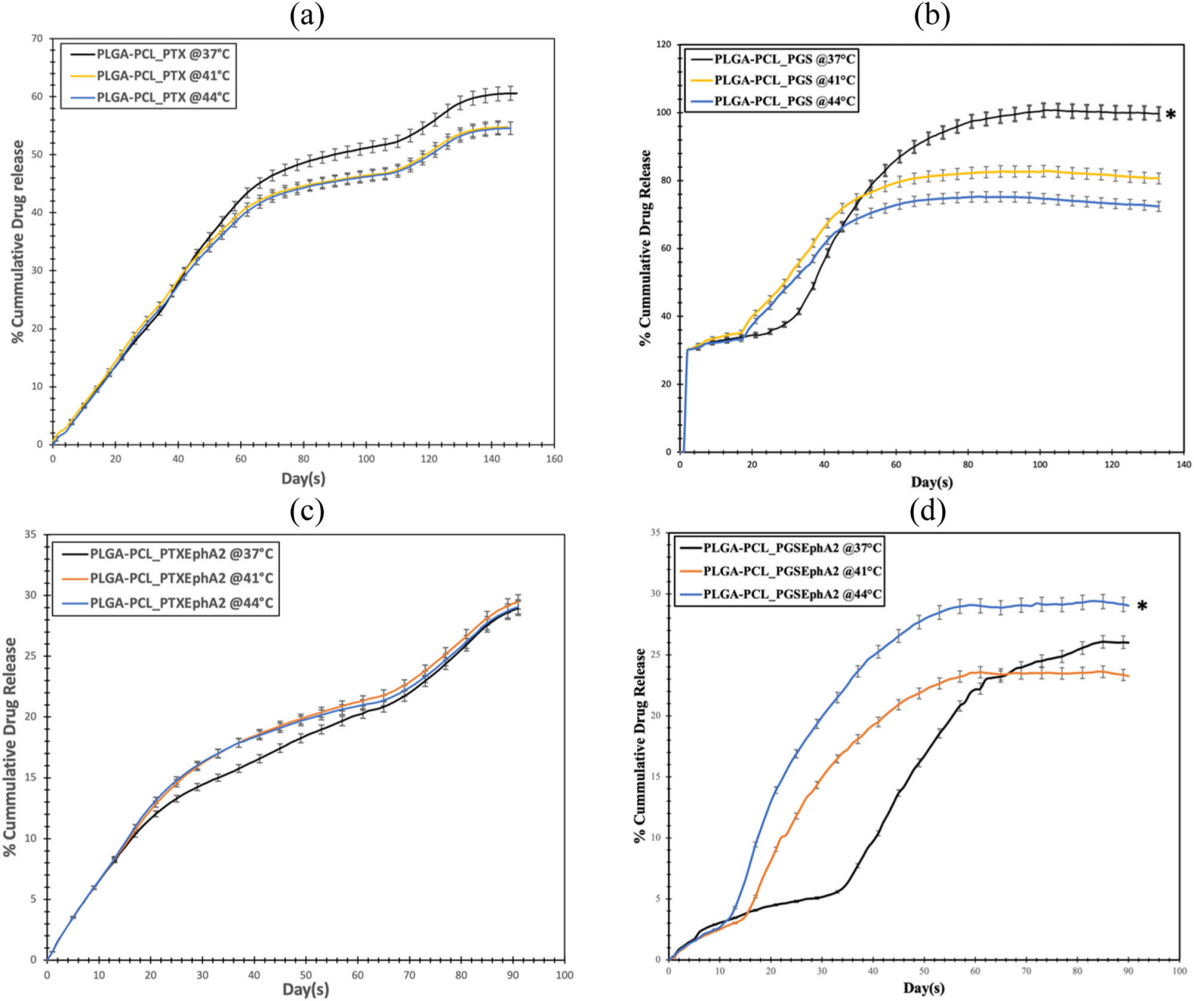
Effect of temperature on the in vitro drug release profile for **a** PLGA-PCL_PTX, **b** PLGA-PCL_PGS, **c** PLGA-PCL_PTXEphA2, and **d** PLGA-PCL_PGSEphA2 drug-loaded microspheres, respectively at 37, 41 and 44 °C. In all cases (*n* = 3, *p < 0.05 vs. control)

Results of in vitro drug release for PLGA-PCL_PTX, PLGA-PCL_PGS, PLGA-PCL_PTXEphA2 and PLGA-PCL_PGSEphA2 microspheres showing percentage of cumulative drug release over time at 37, 41 and 44 °C temperature are presented in Fig. 6a–d. As shown, the sustained drug release behavior of the formulations were studied over a period about 140 and 90 days for targeted and non-targeted encapsulated drugs, respectively, to simulate the physiological in vitro conditions [68, 76].

The drug encapsulation and drug loading efficiency of the targeted drug-loaded microspheres, PLGA-PCL_PTXEphA2 and PLGA-PCL_PGSEphA2 are 94.30%, 78.96% and 4.71%, 3.06%, respectively (See Table 3). These efficiencies are higher than those of the untargeted drug-loaded microspheres formulations, PLGA-PCL_PTX and PLGA-PCL_PGS (77.31%, 2.38% and 65. 91%, 1.96%) as shown in Table 3.

**Table 3:** Drug loading and encapsulation efficiency from the different drug-loaded microspheres formulations

Formulations	Encapsulation efficiency	Drug loading
PLGA-PCL_PTX	77.31	2.38
PLGA-PCL_PGS	94.30	4.71
PLGA-PCL_PTXEphA2	65.91	1.96
PLGA-PCL_PGSEphA2	78.96	3.06

#### Kinetics and thermodynamics of drug release

Table 4 presents summary of drug release kinetics data obtained by fitting the different kinetic models (zero order, first order, Higuchi model and Korsmeyer–Peppas model). From the four different kinetic models used to analyze the PTX- and PGS-loaded polymer formulations, the Korsmeyer–Peppas (K–P) model mostly fit drug release data best. In general, the drug release data in each case of the formulation were better characterized by the K–P model due to the higher correlation coefficients (*R*^2^). The results showed that the K–P model was associated with high correlation coefficients (*R*^2^) between the range of 0.764 and 0.9938.

**Table 4:** Summary of the drug kinetic models showing kinetic constant, release exponent/coefficients and in vitro release constant of the squared correlation coefficient (*R*^2^) for different drug release formulations under body (37 °C) temperature and hyperthermia temperatures (41 °C and 44 °C)

Formulations	Tempt (°C)	Zero order		First order		Higuchi model		Korsmeryer-Peppas		
		*K*	*R*^2^	*K*	*R*^2^	*K*	*R*^2^	*K*	*R*^2^	*n*
PLGA-PCL_PGS	37 °C	0.0283	0.8468	0.0004606	0.4161	2.0004	0.9061	0.6911	0.7641	0.7241
PLGA-PCL_PGSEphA2		0.0144	0.949	0.0013818	0.8804	0.7796	0.8791	0.189	0.9127	0.887
PLGA-PCL_PTX		0.0165	0.9061	0.0006909	0.6474	0.3985	0.9065	0.2081	0.9938	1.0039
PLGA-PCL_PTXEphA2		0.0111	0.9621	0.0009212	0.7004	0.6355	0.9833	0.3546	0.9766	0.7629
PLGA-PCL_PGS	41 °C	0.0179	0.7056	0.0004606	0.268	1.3182	0.8399	0.8004	0.8892	0.679
PLGA-PCL_PGSEphA2		0.0121	0.8378	0.0013818	0.6411	0.6933	0.9055	0.0890	0.9736	1.2327
PLGA-PCL_PTX		0.0154	0.8917	0.0006909	0.6207	1.0929	0.9682	0.2498	0.9919	0.942
PLGA-PCL_PTXEphA2		0.0113	0.9345	0.0009212	0.6662	0.6524	0.983	0.3262	0.9799	0.8048
PLGA-PCL_PGS	44 °C	0.0296	0.9044	0.0004606	0.2209	1.1225	0.8088	0.7435	0.8869	0.6931
PLGA-PCL_PGSEphA2		0.0144	0.8097	0.0012	0.5975	0.8342	0.8961	0.0924	0.9681	1.2636
PLGA-PCL_PTX		0.0154	0.8945	0.0006909	0.6365	1.101	0.9676	0.2151	0.9883	0.9866
PLGA-PCL_PTXEphA2		0.011	0.9258	0.0009212	0.6529	0.6361	0.9787	0.3301	0.9761	0.8009

The kinetic constant (*K*), correlation coefficient (*R*^2^) and Release exponent (*n*) of kinetic data analysis of drug released from the various PLGA-PCL microspheres formulations

**Fig. 7:**
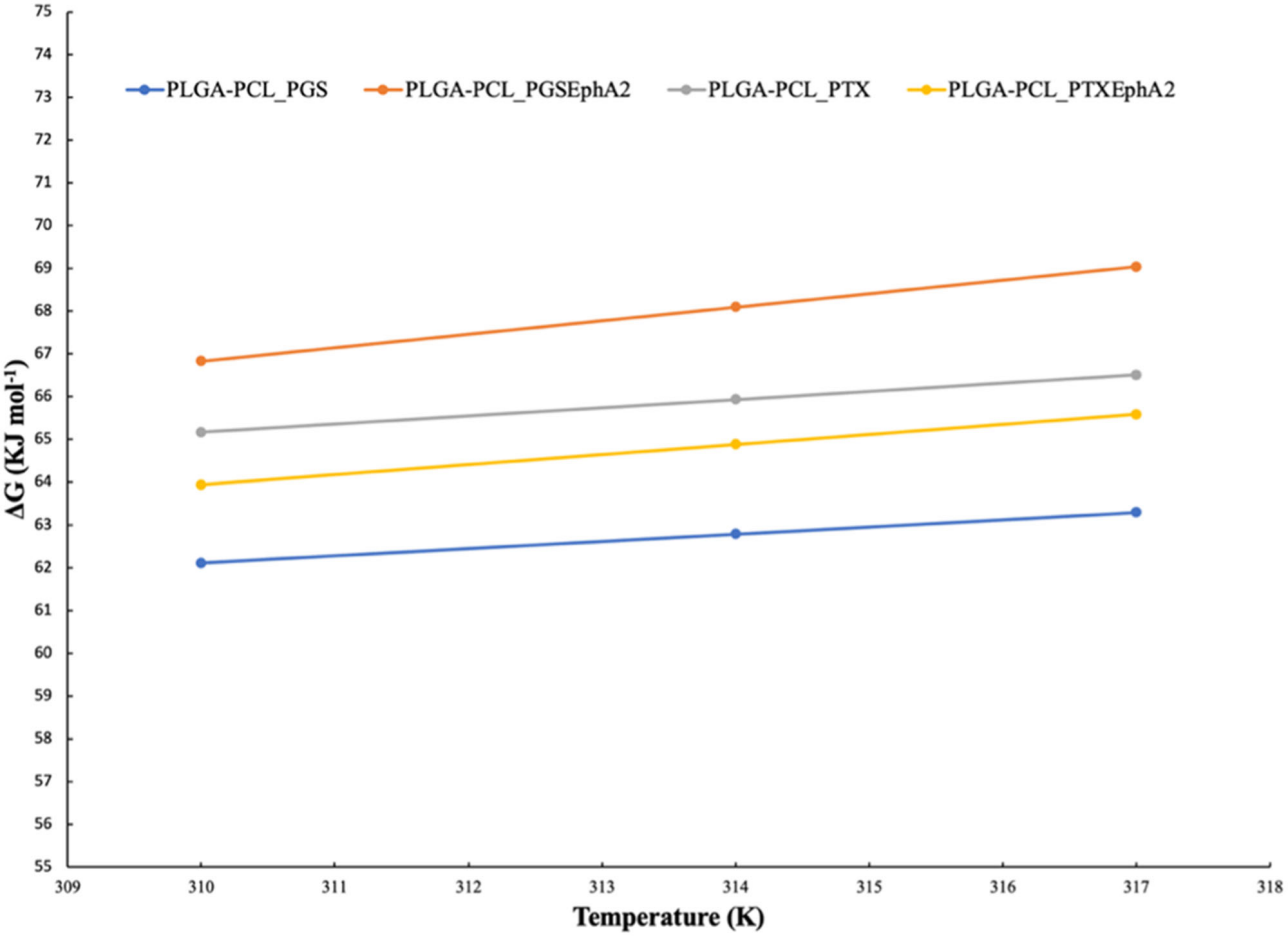
Gibb’s free energy change plot with respect to temperature (K) changes for the various drug-encapsulated PLGA-PCL formulations

The activation energy (*E*_a_), change in enthalpy (Δ*H*), change in entropy (Δ*S*), and Gibb’s free energy change (Δ*G*) (thermodynamics parameters) obtained from the drug release of the drug-loaded PTX-or PGS-drug-based formulations are represented in Table 5. As a follow-up, we plotted the Gibb’s free energy changes with respect to the temperature for the drug release from the drug-loaded microspheres systems (Fig. 7).

**Table 5:** Calculated thermodynamic parameters obtained from PGS and PTX drug release rate from drug-loaded polymer microspheres

Formulations	Temperature (*K*)	*E*_a_ (KJ mol^−1^)	Δ*S* (KJ mol^−1^)	Δ*H* (KJ mo^l−1^)	Δ*G* (KJ mol^−1^)
PLGA-PCL_PGS	310	9.78	−0.17	9.78	62.11
	314				62.79
	317				63.29
PLGA-PCL_PGSEphA2	310	−31.14	−0.32	−31.14	66.83
	314				68.09
	317				69.04
PLGA-PCL_PTX	310	5.80	−0.19	5.80	65.16
	314				65.93
	317				66.50
PLGA-PCL_PTXEphA2	310	−8.86	−0.23	−8.86	63.94
	314				64.88
	317				65.58

#### In vitro degradation of drug-loaded microspheres

**Fig. 8:**
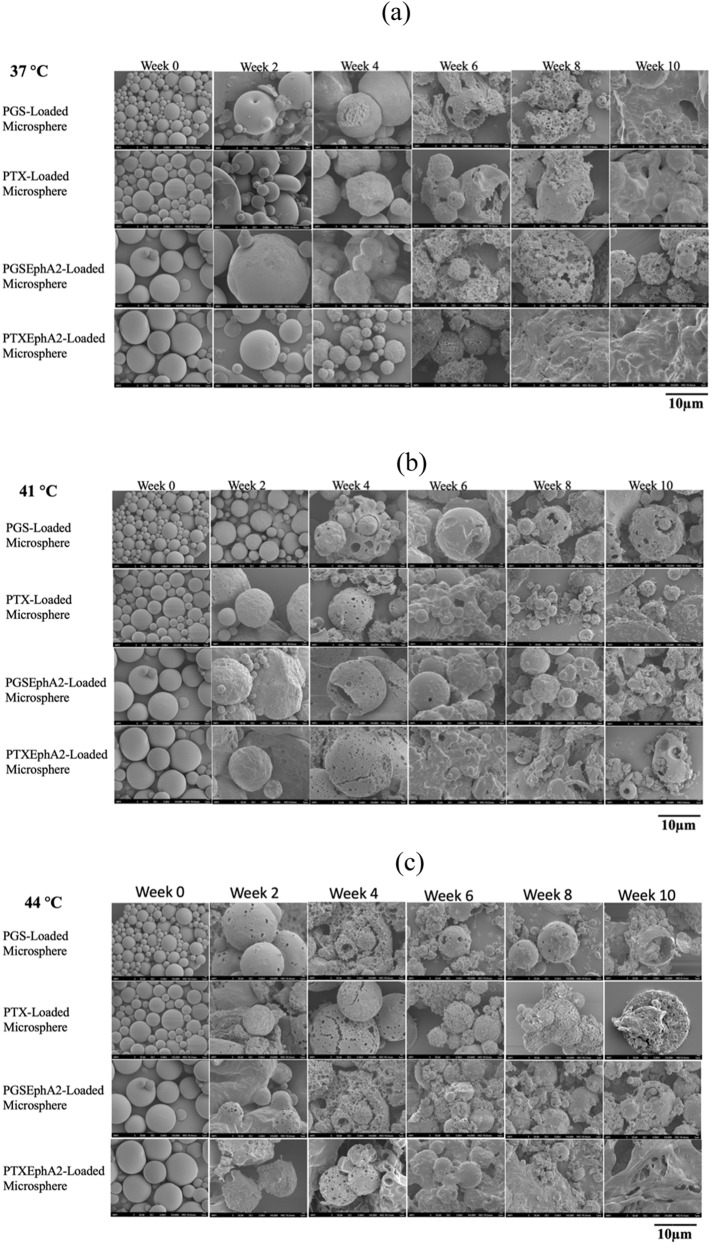
SEM micrographs of drug-loaded PLGA-PCL microspheres for the period of 10 weeks at **a** 37 °C, **b** 41 °C and **c** 44 °C under in vitro conditions in a phosphate buffer saline at pH 7.4. The white arrows show evidence of the progression of material removal and degradation site

To determine and understand the different mechanism of drug release, Fig. 8 shows result of time-dependent sequence of SEM images of degraded drug-loaded microsphere during a period of 10 weeks at the different temperature (37, 41, and 44 °C) of drug release were studied.

### Effect of drug-loaded microspheres on cell viability

**Fig. 9:**
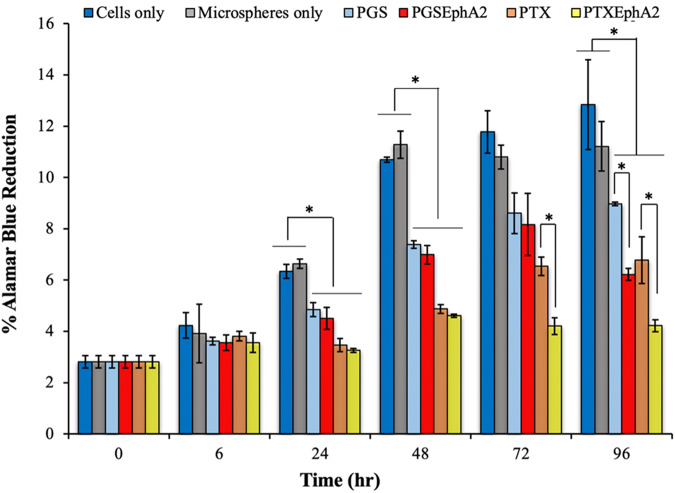
Represents percentage alamar blue reduction for MDA-MB-231 cells exposed to drug-loaded and control PLGA-PCL microspheres at time 6, 24, 48, 72 and 96 h, respectively [*p < 0.05 (*n* = 3)]

The results of the effect of drug from the drug-loaded microspheres on cell viability quantified from alamar blue assay are represented in Fig. 9. The cells viability generally increased with time for the untreated cells (cells only) and the cells treated with microspheres with no drugs loaded (microspheres only). Cell viability was also significantly higher (*p* < 0.05) for the untreated cells and the microspheres only than the cells treated with the microspheres loaded with the drugs (PGS, PGSEphA2, PTX and PTXEphA2).

**Fig. 10:**
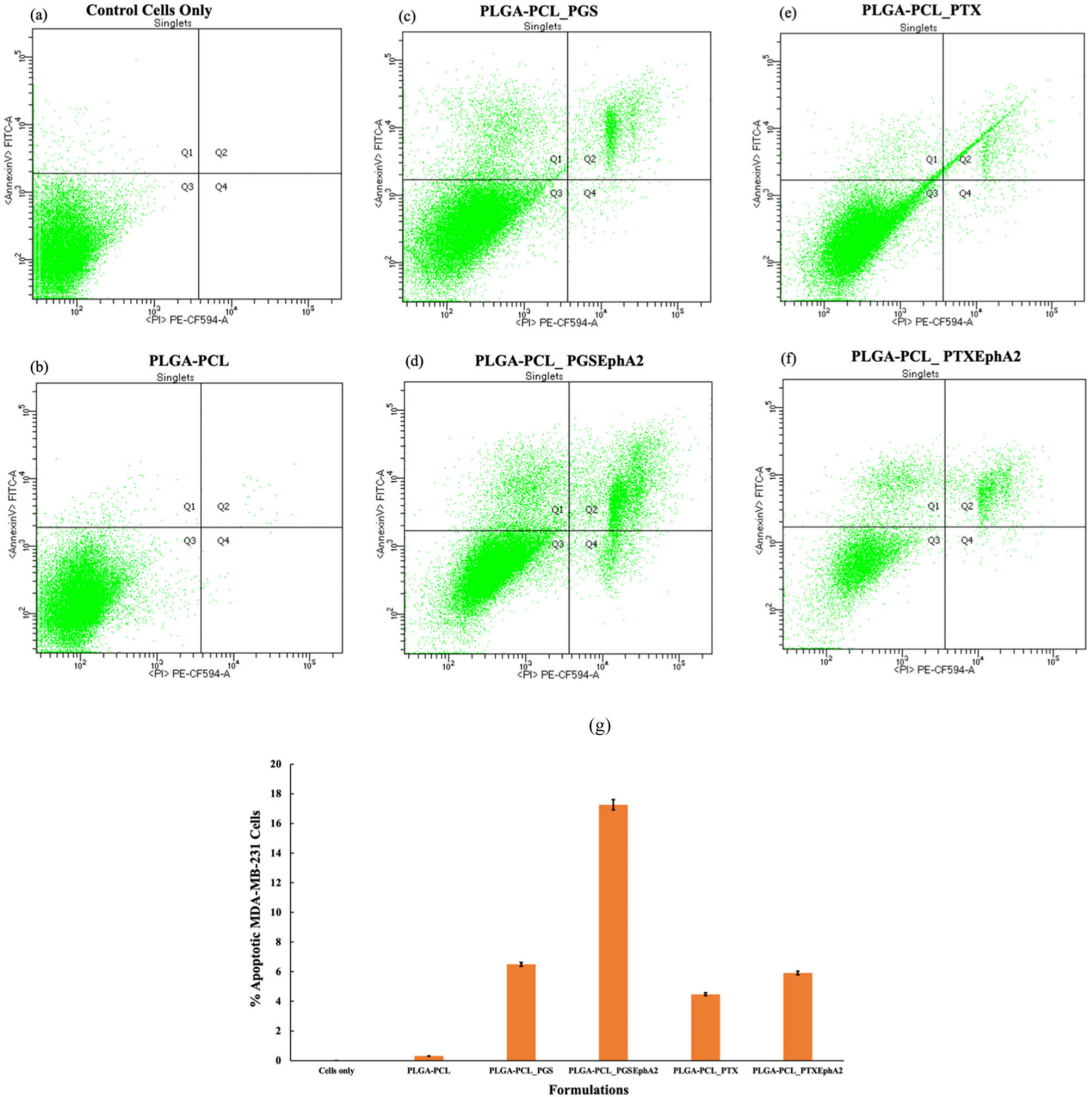
Flowcytometric results showing induction of apoptosis of breast cancer cultured and incubated for 24 h period with **a** control cells **b** PLGA-PCL_PGS **c** PLGA-PCL_PGS **d** PLGA-PCL_PGSEphA2 **e** PLGA-PCL_PTX **f** PLGA-PCL_PTXEphA2 formulations, and their **g** Percentage apoptosis effects of TNBC (MDA-MB-231) cells as a results of anti-tumor drug treatments from the different formulation within 24 h

Results from the flow cytometry (Fig. 10) evaluated the mechanism of cell death by apoptosis or by necrosis from the drug released from the drug-loaded systems. Quadrant 1 - Q1 is equivalent to necrotic cells, Q3 shows live cells with intact membranes, Q4 reveals the early apoptotic cells and Q2 shows the late apoptotic cells.

### In vivo animal studies results

**Fig. 11:**
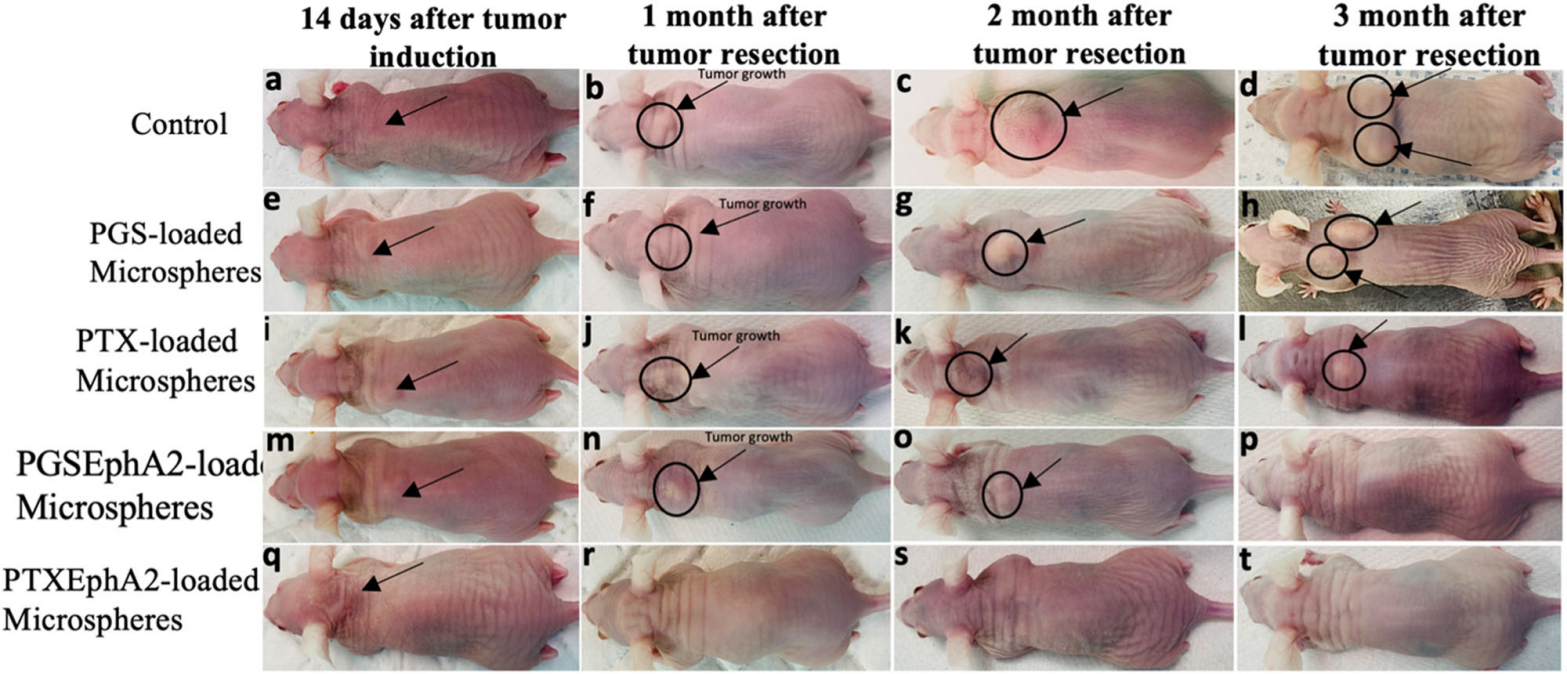
Representative photographs of mouse treatment group showing, (**a**, **e**, **i**, **m**, **q**) 14-days, (**b**, **f**, **j**, **n**, **r**) 1-month, (**c**, **g**, **k**, **o**, **s**) 2-months, and (**d**, **h**, **l**, **p**, **t**) 3-month after treatment with various drug-loaded microspheres after tumor resection of 14-days induced subcutaneous breast cancer tumor

Fourteen days after the induced subcutaneous tumor reached an average tumor volume of ~70 ± 11 mm^3^ as shown in Fig. 11a, e, i, m, q. Interestingly, it was observed that one month after surgical tumor removal (Fig. 11b, f, j, n, r) and implantation of the drug-loaded microspheres formulation, there was still visible regrowth of local regional resected tumors.

### Ex vivo histopathological and cytotoxicity studies

**Fig. 12:**
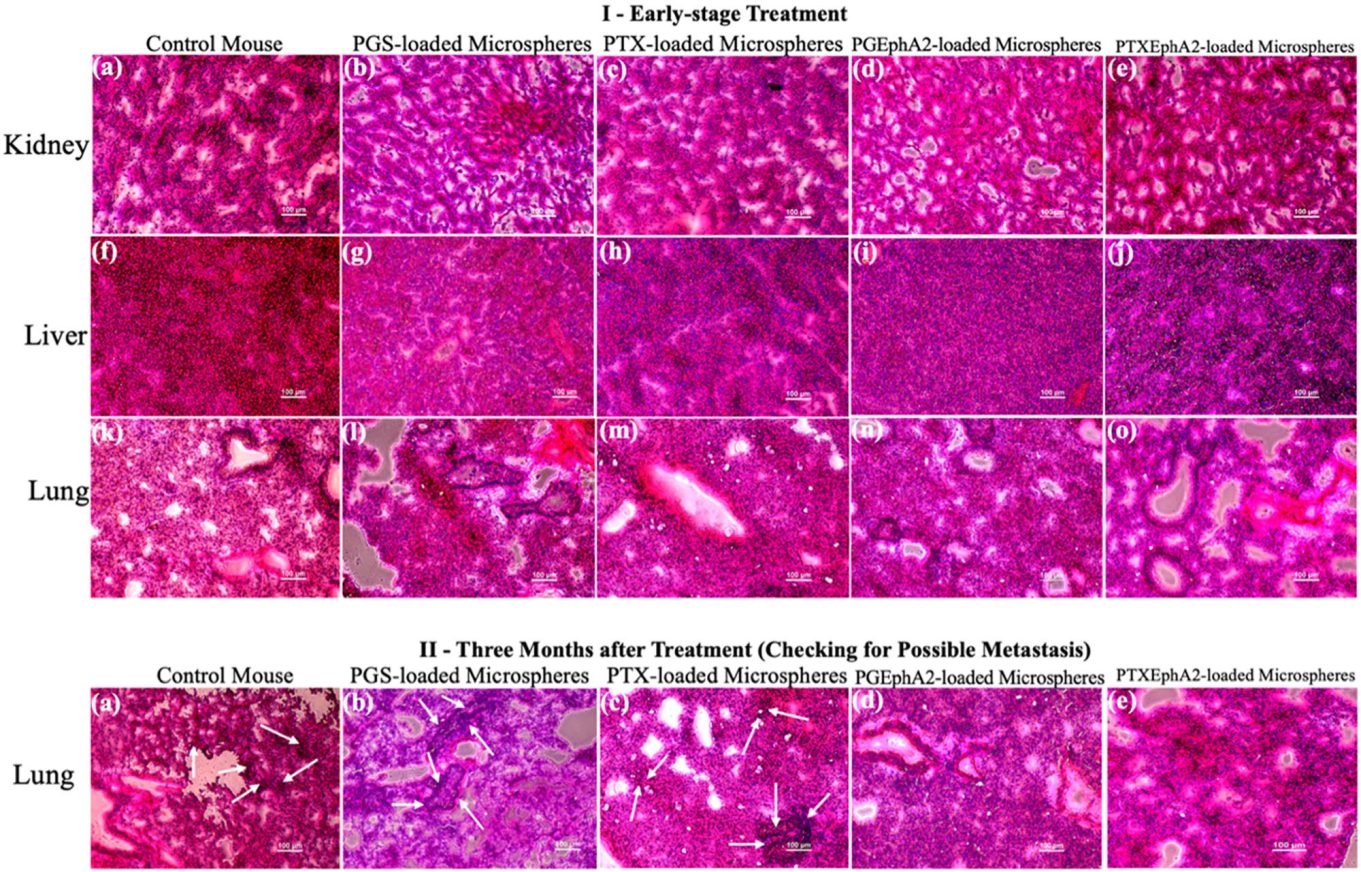
(I) Optical images showing representative H&E histological staining of different early-stage treatment mice groups kidney, liver, and lungs treated with (**a**, **f,** **k**) PLGA-PCL, (**b**, **g**, **l**) PLGA-PCL_PGS, (**c**, **h**, **m**) PLGA-PCL_PTX, (**d**, **i**, **n**) PLGA-PCL_PGSEphA2, and (**e**, **j**, **o**) PLGA-PCL_PTXEphA2 microspheres formulations. (II) Optical images of the (**a**–**e**) lungs treated with PLGA-PCL-control, PLGA-PCL_PGS, PLGA-PCL_PGSEphA2TX, PLGA-PCL_PTXGSEphA2, and PLGA-PCL_PTXEphA2 microspheres formulations

Figure 12 shows representative H&E staining results from treatment mice group treated with drug-loaded microspheres formulations. The results show the effect of the different drugs during the early stage and later stage of treatment on the kidney, liver, and lungs. The goal was to see if there were any significant changes due to cytotoxicity of the drug released from the formulation during the in vivo treatment process. At the early stage of the treatment, we observed that there were no significant histopathological changes in the kidney, liver and lungs of the mice that were treated with the microspheres loaded with the drugs (PGS, PGSEphA2, PTX and PTXEphA2). [See Fig. 12(I)].

Our result showed that 12 weeks after treatment with drug-loaded microspheres, there was evidence of multiple metastatic foci or nodules in the lungs of the control mice, and those treated with PGS and PTX-loaded microspheres formulations [Fig. 12(II)]. However, in the case of mice treated with targeted loaded drugs (PGSEphA2 and PTXEphA2) microspheres formulations, there was no traceable metastasis in the lungs.

## Discussion

### Drug formulations and characterizations

Prodigiosin (PGS) also known as 4-methoxy-5-[(*Z*)- (5-methyl-4-pentyl-2*H*-pyrrol-2-ylidene) methyl]-1*H*,1′*H*-2,2′-bipyrrole that has been shown to significantly reduce the viability with apoptotic effects on breast cancer cells [64, 65, 71, 77–83]. Prodigiosin suppressed the migration of breast cancer MDA-MB-231 and MDA-MB-468 cells in a dose-dependent manner [82].

The presence of the hydrophilic linker (NHS) creates sites for reactions with the methoxy group in the prodigiosin structure. The methoxy group (–OCH_3_) on the PGS also has a high electron density and exhibit a tendency to attack the nucleophilic center of the carbonyl group that is present in the NHS linker [70]. With the presence of EDC, the high electron density attacks the PGS linkages, causing the electrostatic cleavage of the proton from the N–H group, thus linking the EphA2. The reaction with the secondary amine (NH) group creates stable amide linkages with EphA2 that do not easily break down. Thus, in the presence of the EphA2 molecules, NHS ester crosslinks or couples to the ε-amines to the lysine side chains, and to the α-amines in the N-terminals.

In the case of paclitaxel (PTX), the native lysine ε-amines groups of the EphA2-peptide were targeted for the drug coupling. The targeting moieties were attached to PTX via the 2-hydroxyl group (on one of its side chains) in the presence of heterobifunctional linkers. The major function of these linkers is to hold the segment of the drug and EphA2 peptide together sufficiently enough for the ligands to be attached specifically to the target receptors on the cancer cells/tumor. Therefore, the presence of characteristic bands of –NH_2_ (3300 cm^−1^) and amide (covalent or peptide) bond signatures at around 1610 cm^−1^ from the FTIR peaks in Fig. 1, indicate that the drugs were successfully conjugated [72].

Results from our formulation showed in Table 1 shows a decreasing microspheres yield considering the theoretical amount of blend of polymer systems used as starting materials. The decrease in yield can be attributed to the process of microsphere synthesis which includes sonication, washing out the surfactant and the process of drying using the lyophilizer, all these led to the loss of polymers.

Our study revealed that the different drug-encapsulated microspheres were spherical in shape with unique outer surface. Based on the architecture and the method of preparation, some drug particles may have been distributed across the polymer microspheres. Despite the variation in the sizes of the microparticles, our results showed that there are no significant morphological differences between the drug-encapsulated PLGA-PCL microspheres and the control PLGA-PCL microspheres. Therefore, based on the sizes, our results suggested that the presence of drug in the different polymer-based formulations did not significantly affect the sizes and morphologies of the targeted drug-encapsulated microparticles. For the mean hydrodynamic bar chart, we can clearly observe that the mean hydrodynamic diameters (particle sizes) from DLS were greater than the mean particles sizes obtained from SEM characterization (Fig. 3). The differences are attributed to the adsorption or swelling nature of the polymer system in the DLS medium [84].

The FTIR spectra obtained for the drug-loaded PLGA-PCL microspheres were like those of the control PLGA-PCL microspheres (Fig. 4). The drug-loaded microspheres (PLGA-PCL_PGS, PLGA-PCL_PTX, PLGA-PCL_PGSEphA2 and PLGA-PCL_PTXEphA2) showed similar spectra compared to the PLGA-PCL microspheres spectrum. This indicates that there was no modification on the chemical groups of PLGA and PCL due to drug loading. There is a characteristic band formed at 2944 cm^−1^ which can be associated to the stretching of CH, CH_2_ and CH_3_ groups, a peak at 1750 cm^−1^ was due to the stretching of the C=O bond in the lactide and glycolide structure. The peak at 1187 cm^−1^ was attributed to the stretching of C–O and C–C bonds associated with PLGA and PCL. The peak formed at 732 cm^−1^ is attributed to C–H bending of the polymer blend (Fig. 4a).

The DSC thermographs in Fig. 4b show that the glass transition, Tg and melting (endothermic peak) temperatures, Tm of PLGA-PCL increased slightly in the presence loaded drug and the change in heat capacity, ∆Cp decreased in the presence of encapsulated drug loading as shown in Table 2. The increase in Tg in the drug-loaded systems, may have been responsible in the decrease of polymer mobility via thermal induced structural relaxation of polymer in a way that reduces the rate of diffusion and promotes sustain release of the drug from the encapsulated drug polymer system [85]. This suggests that the drugs are reasonably miscible with the blend of polymers for the control and localize release [86]. The DSC helps to investigate the physical state of the dispersed drug in the drug-encapsulated microspheres. Figure 4c graphically displays the thermogravimetric analysis of the PLGA-PCL control and the drug-loaded microspheres. Also, the mass % and temperature profile indicated that the polymers remained the same until the temperature got to 300 °C. The results show that the thermal decomposition of PLGA-PCL, PLGA-PCL_PGS, PLGA-PCL_PGSEphA2, PLGA-PCL_PTX and PLGA-PCL_PTXEphA2 occurred at temperatures 325.2, 304.3, 313.7, 318.8, and 320.5 °C respectively. The result indicates that the decomposition temperatures of PLGA-PCL microspheres in the presence of encapsulated drug.

The HNMR spectra obtained for the PLGA- PCL and drug-loaded PLGA-PCL microspheres formulations were all similar as shown in Fig. 5 with six principal peaks (ppm). The deuterated chloroform used as a solvent was observed by the chemical shift at 7.26 ppm, this peak in the spectra suggests that the solvent was successfully incorporated and can serve as a reference peak [87]. The HNMR spectra results suggest that the blend of polymers did not undergo chemical modification during drug loading and encapsulation.

### In vitro drug release kinetics, thermodynamics and degradation

The results of the in vitro drug release are relevant because typically the chemotherapy treatment for triple negative breast cancer takes between three and six months [66]. This suggestion has guided our efforts in the design of these targeted drug-encapsulated blend of microspheres formulations in a way that we critically studied the release profile, microspheres degradation, kinetics, and thermodynamics of the drugs from the formulations within the treatment relevant period.

Interestingly, for each of the formulations under different temperatures, the release profiles are similar. The initial drug release (burst release) in the first 20 days was unique for the PGS-based formulations (PLGA-PCL_PGS and PLGA-PCL_PGSEphA2) as compared to PTX-based drug-loaded systems (Fig. 6). This was attributed to the hydrophilic and hydrophobic moieties in the PGS-based drugs [65]. However, in all cases, there are very interesting three stages drug release from the release curve profile. From the drug release profile, the three stages may be attributed to diffusion, dissolution, and degradation in the presence of the blend of polymers used for the encapsulation.

The drug release under different temperatures carried out does not significantly influence profile in the case PTX-based drug-loaded systems (Fig. 6a, c). However, in the case of PGS-based drug-loaded formulations, temperature variation influenced the release profile. Higher temperature resulted in an increase in higher cumulative drug release (Fig. 6b, d). Overall, the percentage cumulative for PLGA-PCL_PTX, PLGA-PCL_PGS, PLGA-PCL_PTXEphA2, and PLGA-PCL_PGSEphA2 is ~60, 98, 28, and 27%, respectively. Clearly, the unconjugated-drug-loaded systems have higher percentage cumulative release than those of the EphA2-conjugated microspheres formulations.

The results in Table 3 indicates that, the increase of drug encapsulation and drug loading encapsulation efficiency in the targeted drug-loaded microspheres may be attributed to presence of hydrophobic–hydrophilic moieties and nature of the conjugated drug (PTXEphA2 and PGSEphA2) used [65]. In general, our results reveal that the encapsulation and drug loading efficiencies of PTX-based drug-loaded formulations (PLGA-PCL_PTX and PLGA-PCL_PTXEphA2) were greater than those of PGS-based drug-loaded systems (PLGA-PCL_PGS and PLGA-PCL_PGSEphA2).

Usually, there is a need to be aware of the relationship of concentrations used in a preclinical versus clinical settings. It is believed that this understanding will provide relevant insights for translational research [88]. Therefore, it is crucial to note that the concentration adopted for this work is within a limit relevant for clinical study when conversion factor from humans subject to mice and cells is considered as approved for cancer treatment by the FDA [88].

In the case of in vitro drug release kinetics and thermodynamics, the drug release exponents, n, obtained for each of the released drug PTX, PTXEphA2, PGS, and PGSEphA2 from their respective microsphere formulations were > 0.5. Based on the drug release exponent ‘n’ obtained, majority of the formulations fall within 0.679–0.887 range characterizing the release as an anomalous diffusion or non-Fickian diffusion. The range of “n” is consistent with drug release by anomalous transport or non-Fickian diffusion that has been shown to involves two phenomena (drug diffusion and relaxation of the polymer matrix) [89]. However, there were some cases where “n” value ranges from 0.942–0.986 (Table 4) that is characterized as case 2-relaxation or non-Fickian case 2. Finally, there were very few cases where we observed non-Fickian super case 2 because the ‘n’ value was greater than 1. The anomalous nature of the drug release profile obtained may be due to the blend of PLGA-PCL polymer material and the temperature variation which may have led to induce reservoir effects, leading to sustained release of the PGS or PTX drugs.

Interestingly, the PGS- and PTX-loaded polymer microspheres have ∆*H* > 0. This show that the results of the drugs (PGS and PTX) release occur by endothermic process [90]. On the contrary based on the thermodynamics enthalpy parameter, the drugs (PGSEphA2 and PTXEphA2) release from the PGSEphA2- and PTXEphA2-loaded polymer microspheres occur by exothermic process because ∆*H* < 0. This may not be unrelated to the conjugated drugs and why the cumulative drug release is low. Table 5 show all negative values for entropy change (ΔS). These ΔS show that there is a decrease in the disorder with increasing drug release from the various drug-loaded PLGA-PCL microspheres [64, 90]. Finally, from Fig. 7 and Table 5 the Gibb’s free Energy changes are all greater than zero (∆*G* > 0). Therefore, ∆G values for all the drug-loaded PLGA-PCL microsphere formulations are positive. The results revealed that drug release process is non-spontaneous and feasible. The non-spontaneous nature of the drug release may be due to controlled release over a period [91].

From the drug-loaded degradation results shown, it was observed that after week 2 of drug release at 37 °C, the microspheres surfaces were still smooth with traces of pores formulation. It is believed that during this phase, the release of drugs was mainly through diffusion. For the hyperthermic temperatures studied (41 and 44 °C), the microsphere surfaces were rough and had bigger pores developing as compared to those studied at 37 °C. Our results showed that degradation increases as the temperature increases from 37, 41, and 44 °C. These results explain why there was increase in drug release from the blend of microspheres as in vitro drug release temperature increases.

Consequently, there were gradual morphological changes observed in the structure microspheres. These changes form rougher drug microsphere surfaces with larger pore sizes, increasing agglomeration coupled with loss of sphericity. Early degradation may be due to hydrolytic degradation of the polymer microspheres which initially start as surface erosion [92]. In the later stages of degradation, there was evidence of erosion observed in the structure of the microspheres due to evidence of pit collapse of the drug-loaded polymer structures. In the later stage, the degradation of the drug-loaded microspheres is consistent with prior results of degradation-induced morphological changes of drug-loaded blends polymer microspheres from bulk erosion to surface erosion [64, 92]. The increased erosion observed after week 8 is attributed to the hydrolytic degradation of the polymer ester and drug leaching [93].

The presence of PLGA in each of the different formulations may have been responsible in enhancing the degradation by moderating the hydrophilicity of the drug-loaded blend of the microspheres during the period of drug release [94]. The PCL in the blend of drug-loaded PLGA-PCL is thought to play a vital role in mass loss of PLGA-PCL which in turns has implications in the sustained release of the drugs [95]. These drug release results from degradation create may be the reasons why the PLGA-PCL drug-loaded microspheres have robust characteristics for longer-lasting and controlled release of drugs within a safe regime that is relevant and needed for clinical settings [65, 95].

### Drug effects from drug-encapsulated microspheres

The results from the alamar blue assay critically helped to understand the effect of the drug eluted from the drug-loaded formulations by quantifying the cell metabolic activity and the percentage alamar blue reduction. A higher percentage alamar blue reduction corresponded to a greater cell viability. A higher percentage alamar blue reduction corresponded to a greater cell viability. This assay was used to assess the effects of the different drugs released from the drug-loaded microspheres on cell viability. The results (Fig. 9) showed that there was no significant difference between the viabilities of untreated cells and cells treated microspheres (*p* > 0.05) at 6 h period. Among the drug-loaded microspheres, cells treated with the microspheres loaded with EphA2- conjugated prodigiosin (PGSEphA2) had less viable cells after 72 and 96 h (*p* < 0.05) than those of the unconjugated prodigiosin (PGS). Similarly, the EphA2-conjugated paclitaxel (PTXEphA2) loaded microspheres reduced the viability of the MDA-MB_231 breast cancer cells than the unconjugated paclitaxel (PTX)-loaded microspheres (*p* < 0.05) after 72 and 96 h. In general, the results showed that the specificity of the conjugated drug from the formulations (PLGA-PCL_PTXEphA2, and PLGA-PCL_PGSEphA2) helped to reduced cell viability of the TNBC as compared to the unconjugated drug.

The results from Fig. 10 show different cell apoptotic distributions for breast cancer cells (MDA-MB-231) in the presence of different drug-loaded microspheres. The density plots obtained 24 h after incubating cells with the different drug-encapsulated microspheres from the flow cytometry suggests that all the drug-encapsulated systems used induced majorly induced late-stage apoptosis (in quadrant 2 - Q2). Although it is noted that a few populations of cancer cells exist in necrotic stages where their cell membranes are damaged.

However, PGSEphA2 drug from PLGA-PCL_PGSEphA2 seem to have higher apoptotic effect followed by PTXEphA2 from PLGA-PCL_PTXEphA2, PGS from PLGA-PCL_PGS, and PTX from PLGA-PCL_PTX [Fig. 10 (g)]. This outcome may be unrelated to drug release data and burst release result. The results also suggest that drug-conjugated drug formulations (PLGA-PCL_PGSEphA2 and PLGA-PCL_PTXEphA2) are specific in targeting the cancer and thereby have higher apoptotic effect on TNBC than the unconjugated drugs formulations (PLGA-PCL_PGS and PLGA-PCL_PTX). In general, the targeted encapsulated drugs in the microsphere’s formulations may be immobilized due to their specificity in a way that when in contact with breast cancer cells they become susceptible to localized growth inhibition.

### In vivo studies

Results after implantation of drug-loaded microspheres in the presence of control, first we observed that control mice without drug show recurrences of tumor that is attributed to the incomplete removal of all the residual tumor and the absence of drug-loaded microspheres. However, while these local regionals tumors keep growing in the case of PGS- and PTX-loaded microspheres formulation like the control tumors after two months of tumor removal [Fig. 11c, g, k, o, s], there were almost complete elimination of the tumor for PGSEphA2- and PTXEphA2-loaded microspheres formulations.

After three months of tumor resection and implantation of drug-loaded systems, it was clearly observed that the targeted drug-loaded formulation (PGSEphA2- and PTXEphA2-loaded microspheres) eliminated completely the local regional tumors [Fig. 11p, t]. However, we observed multiple regrowth of local regional tumors in the case of the control mice and mice treated with unconjugated-drug-loaded systems (PGS- and PTX-loaded microspheres) (See Fig. 11d, h, i). In generally, there no recurrence of tumor observed on mice treated with targeted microsphere’s formulation (PGSEphA2- and PTXEphA2-loaded microspheres) after 12 weeks of localized drug release. This was due to the combination of the specificity of the targeted drug coupled with the localization of control drug release.

The results from the weight of the mice measured during the therapeutic process of the in vivo experiments, clearly show that there were no significant changes in the body weight of the mice treated with drug-loaded microspheres and those of the control group. The results imply that all the categories of drug-loaded microspheres used did not create any cytotoxic effects on the general well-being of the group of mice used for the treatment during the therapeutic time. Evidence was seen in the normal eyes, skin and fur of the mice implanted with the drug-loaded systems. Although there was a slight increase observed in body weight of the treatment groups, we observed that this increase in body weight was synonymous to the control group. Thus, validating our observation that there were no noticeable physiological changes, drastic decrease in the body weight or side effects after the administration of the drug-loaded microspheres, when compared to the control mice [65, 71].

### Ex vivo histopathological studies

At the early stage, the absence of noticeable histopathological changes in the kidney, liver and lungs of the control mice and those that were treated with the drugs (PGS, PGSEphA2, PTX and PTXEphA2). There was no evidence of pulmonary edema or hyperplasia or liver cell hyaline degeneration and necrosis [71]. The glomerular volume of the kidneys was observed to be normal with no evidence of renal hyperplasia [71].

However, 12 weeks after treatment with drug-loaded microspheres, the result validated that the targeted drugs (PGSEphA2 and PTXEphA2) in the microspheres formulations are specific and effective for the targeting and elimination of TNBC. This outcome is due to the specificity of the drug which was confirmed in the in vitro.

### Implications

The implications of this work are significant and will pave way for the design of targeted drug-encapsulated microspheres formulations for the specific and localized treatment of TNBC. Using a blend of PLGA and PCL tends to regulate and prolong the release of drug from encapsulated polymer systems [58] for a period of over 90 days. Interestingly, the drug released kinetics, thermodynamics and degradation of the drug-loaded PLGA-PCL microspheres were critically studied. The drug release of the anticancer drugs (PGS, PTX, PGSEphA2 and PTXEphA2) is tri-phasic in nature, and mainly characterized by anomalous (non-Fickian) diffusion. The mechanism of drug release is characterized by diffusion, dissolution, and degradation of the drug-loaded systems. The in vitro release of the targeted drug at the body temperature has been shown to reduce the viability of TNBC cells (MDA-MB-231). Such unique delivery facilitates new insights that provides new opportunities in the development of unique injectable or implantable targeted drug-based polymer microspheres formulations for treatment and prevention of local recurred triple negative breast cancer after tumor surgery.

Finally, we envisage a potential of using well-characterized microsphere as an implantable or injectable targeted drug-encapsulated formulation as therapeutic vehicle for variety of treatment applications that require controlled and localized drug delivery [56, 96]. For example, these drug-loaded microparticle formulations can be relevant for the treatment of solid tumors of breast, prostate, and cervical cancers that have been shown to overexpress specific types of receptors. The treatment can be achieved via localized elution of drugs within local or into surrounding tissue at a controlled rate without any significant side adverse effects as compared to bulk chemotherapy treatment.

## Summary and concluding remarks

Targeted drug-loaded blend of PLGA-PCL microparticles developed are promising therapeutic formulations that are characterized with unique and sustained control drug release. Breast cancer overexpressed EphA2-conjugated PGS and PTX encapsulated microspheres have shown increased efficiency in the treatment of breast cancer due to their specificity and overexpression on the surfaces of TNBC. Under in vitro and in vivo conditions, our results suggest that the specific targeting is enhanced by increased adhesion of the EphA2-conjugated drugs to TNBC cells/tissues under in vitro and in vivo studies [32]. The EphA2-conjugated drugs (PGSEphA2 and PTXEphA2) also increase the inhibition of MDA-MB-231 TNBCs more than the unconjugated drugs (PGS and PTX).

Furthermore, the ex vivo histopathological results revealed no evidence of physiological changes due to localized treatment of TNBC with EphA2-conjugated drug. The targeted drug-loaded PLGA-PCL formulation was effective in targeting and treatment that those of drug-loaded PLGA-PCL formulation. Interestingly, there were no adverse differences in mortality, or changes in body weight that were observed as compared to control mice after treatment with PGSEphA2- or PTXEphA2-loaded microspheres formulation. This suggests that the proliferation of the induced xenografts TNBC tumors in the athymic nude mice was specifically and robustly inhibited by PGSEphA2 or PTXEphA2. Hence, the current results show that EphA2-conjugated PGS and EphA2-conjugated PTX significantly enhance the specific targeting and localized treatment of TNBCs without adverse toxicity effects. Therefore, the prolonged and controlled delivery of EphA2-conjugated prodigiosin (PGS) and paclitaxel (PTX) from PLGA-PCL-based microspheres has great potential to prevent the regrowth or locoregional recurrence of TNBC after surgical resection. Although, the focus of this study is to explore localized and targeted release of drug from drug-loaded microspheres under in vitro microenvironment that mimics the body pH (7.4) and in vivo studies for the treatment of breast cancer, further work is needed to study the potential effect of pH of the in vitro release and in vivo microenvironment.
